# Repression of Human Papillomavirus Oncogene Expression under Hypoxia Is Mediated by PI3K/mTORC2/AKT Signaling

**DOI:** 10.1128/mBio.02323-18

**Published:** 2019-02-12

**Authors:** Felicitas Bossler, Bianca J. Kuhn, Thomas Günther, Stephen J. Kraemer, Prajakta Khalkar, Svenja Adrian, Claudia Lohrey, Angela Holzer, Mitsugu Shimobayashi, Matthias Dürst, Arnulf Mayer, Frank Rösl, Adam Grundhoff, Jeroen Krijgsveld, Karin Hoppe-Seyler, Felix Hoppe-Seyler

**Affiliations:** a Molecular Therapy of Virus-Associated Cancers, German Cancer Research Center (DKFZ), Heidelberg, Germany; b Faculty of Biosciences, Heidelberg University, Heidelberg, Germany; c Division of Proteomics of Stem Cells and Cancer, German Cancer Research Center (DKFZ), Heidelberg, Germany; d Heinrich Pette Institute, Leibniz Institute for Experimental Virology, Hamburg, Germany; e Division of Theoretical Bioinformatics, German Cancer Research Center (DKFZ), Heidelberg, Germany; f Viral Transformation Mechanisms, German Cancer Research Center (DKFZ), Heidelberg, Germany; g Biozentrum University of Basel, Basel, Switzerland; h Department of Gynaecology, Jena University Hospital, Jena, Germany; i Department of Radiooncology and Radiotherapy, Mainz University Medical Center, Mainz, Germany; j Medical Faculty, Heidelberg University, Heidelberg, Germany; Tufts University School of Medicine; Icahn School of Medicine at Mount Sinai

**Keywords:** AKT, cervical cancer, human papillomavirus, tumor virus

## Abstract

Hypoxia is linked to therapeutic resistance and poor clinical prognosis for many tumor entities, including human papillomavirus (HPV)-positive cancers. Notably, HPV-positive cancer cells can induce a dormant state under hypoxia, characterized by a reversible growth arrest and strong repression of viral E6/E7 oncogene expression, which could contribute to therapy resistance, immune evasion and tumor recurrence. The present work aimed to gain mechanistic insights into the pathway(s) underlying HPV oncogene repression under hypoxia. We show that E6/E7 downregulation is mediated by hypoxia-induced stimulation of AKT signaling. Ablating AKT function in hypoxic HPV-positive cancer cells by using chemical inhibitors efficiently counteracts E6/E7 repression. Isoform-specific activation or downregulation of AKT1 and AKT2 reveals that both AKT isoforms contribute to hypoxic E6/E7 repression and act in a functionally redundant manner. Hypoxic AKT activation and consecutive E6/E7 repression is dependent on the activities of the canonical upstream AKT regulators phosphoinositide 3-kinase (PI3K) and mechanistic target of rapamycin (mTOR) complex 2 (mTORC2). Hypoxic downregulation of E6/E7 occurs, at least in part, at the transcriptional level. Modulation of E6/E7 expression by the PI3K/mTORC2/AKT cascade is hypoxia specific and not observed in normoxic HPV-positive cancer cells. Quantitative proteome analyses identify additional factors as candidates to be involved in hypoxia-induced activation of the PI3K/mTORC2/AKT signaling cascade and in the AKT-dependent repression of the E6/E7 oncogenes under hypoxia. Collectively, these data uncover a functional key role of the PI3K/mTORC2/AKT signaling cascade for viral oncogene repression in hypoxic HPV-positive cancer cells and provide new insights into the poorly understood cross talk between oncogenic HPVs and their host cells under hypoxia.

## Abstract (precis)

Oncogenic HPV types are major human carcinogens. Under hypoxia, HPV-positive cancer cells can repress the viral E6/E7 oncogenes and induce a reversible growth arrest. This response could contribute to therapy resistance, immune evasion, and tumor recurrence upon reoxygenation. Here, we uncover evidence that HPV oncogene repression is mediated by hypoxia-induced activation of canonical PI3K/mTORC2/AKT signaling. AKT-dependent downregulation of E6/E7 is only observed under hypoxia and occurs, at least in part, at the transcriptional level. Quantitative proteome analyses identify additional factors as candidates to be involved in AKT-dependent E6/E7 repression and/or hypoxic PI3K/mTORC2/AKT activation. These results connect PI3K/mTORC2/AKT signaling with HPV oncogene regulation, providing new mechanistic insights into the cross talk between oncogenic HPVs and their host cells.

## INTRODUCTION

Human papillomaviruses (HPVs) are small double-stranded DNA viruses. They are grouped into low-risk and high-risk types according to their tumorigenic potential. High-risk HPV types are of high medical relevance in that they are closely linked to common oropharyngeal and anogenital cancers (1), accounting for approximately 4.5% of the total cancer incidence in humans (2). Prophylactic vaccination has proven to be effective in preventing infection with the most common oncogenic HPV types (3). However, cervical cancer is expected to remain a significant global health burden for many years (4, 5), since worldwide vaccination rates are still disappointing and especially low in developing countries, where cervical cancer incidence is particularly high (6). Moreover, the available prophylactic vaccines will not prevent cancer progression in already persistently infected persons, a process which typically occurs over decades (4, 5). Thus, there is an urgent need for the development of novel therapies, which should benefit from a better understanding of the biology of HPV-associated cancers.

The growth of HPV-positive tumor cells is considered to depend on the sustained expression of the two viral oncogenes E6 and E7 (1, 5, 7), since silencing of E6/E7 expression rapidly induces cellular senescence, an irreversible growth arrest (8–11). Recently, however, it was uncovered that E6/E7 expression is strongly, but reversibly, downregulated in cervical cancer cells under hypoxia, and yet, induction of senescence is severely impaired (11). Instead, hypoxic cervical cancer cells enter a dormant state, characterized by a reversible growth arrest. Upon reoxygenation, the cells reactivate E6/E7 expression and resume proliferation (11). The dormant state of hypoxic HPV-positive cancer cells may support immune evasion, therapy resistance, and tumor recurrence (11) and thereby could contribute to the poor clinical prognosis associated with tumor hypoxia (12–14). Moreover, these findings indicate the existence of a hitherto unknown mechanism by which cervical cancer cells efficiently shut down viral oncogene expression under hypoxic conditions.

The serine-threonine kinase AKT is part of the phosphoinositide 3-kinase (PI3K)/AKT signaling cascade, which plays a key role in orchestrating the cellular response to various external and internal stimuli and, consequently, is involved in the regulation of diverse cellular functions, such as proliferation, survival, and metabolism (15). The PI3K/AKT pathway is aberrantly activated in a variety of cancers and contributes to the development and maintenance of tumors and to their resistance toward standard therapies. Hence, this pathway is a promising target for cancer therapy and is currently the focus of many clinical studies using PI3K/AKT inhibitors as single agents or in combination with conventional therapies (16). Cervical cancer patients also show frequent dysregulation of PI3K/AKT activity and, thus, are subject to PI3K/AKT-targeting clinical trials (17–19).

Notably, AKT phosphorylation and signaling can also be activated by hypoxia (20–22). Here, we show that the inhibition of E6/E7 expression in hypoxic cervical cancer cells is mediated by the hypoxia-induced activation of the PI3K/mechanistic target of rapamycin (mTOR) complex 2 (mTORC2)/AKT pathway. E6/E7 repression is conferred by the AKT1 and AKT2 isoforms, which act in a functionally redundant manner, ultimately leading to the inhibition of the *E6/E7* promoter. Hypoxia-linked E6/E7 repression is glucose sensitive and can be counteracted by PI3K/mTORC2/AKT inhibitors. Proteome analyses under these different experimental conditions identified several cellular proteins which potentially represent additional upstream or downstream factors involved in hypoxic AKT activation and E6/E7 oncogene repression.

## RESULTS

### Hypoxia induces AKT phosphorylation that inversely correlates with E6/E7 expression in a glucose-sensitive manner.

To gain insights into the molecular mechanisms underlying the hypoxic repression of E6/E7, we investigated candidate pathways known to be important in the cellular adaptation to hypoxia. Since we previously found no indication for a role of the hypoxia-inducible factors HIF-1α and HIF-2α in E6/E7 repression (11), we examined another master regulator of hypoxia-responsive gene regulation, the repressor element 1-silencing transcription factor (REST), which is, contrarily to HIF, mainly involved in transcriptional repression (23). However, inhibition of REST expression by small hairpin RNA (shRNA) did not affect the hypoxic downregulation of E6/E7 (Fig. S1 in the supplemental material), arguing against a role of REST in this process. Since hypoxic repression of E6/E7 is glucose sensitive, in that it is diminished when cells are cultured in medium containing unphysiologically large amounts of glucose (25 mM) (11), we also investigated the ChREBP/MondoA-Mlx transcription factors. These are major orchestrators of glucose-dependent gene regulation, and their activity can be modulated under hypoxia, also in an HIF-independent manner (24, 25). However, we found no evidence connecting Mlx, the obligatory dimerization partner of ChREBP and MondoA (25), to E6/E7 expression in knockdown experiments (Fig. S2).

10.1128/mBio.02323-18.1 FIG S1 REST expression is not linked to E6/E7 repression under hypoxia. HeLa cells expressing shRNAs targeting REST (shREST) or a control shRNA (shContr-1) were cultured for 24 h at the indicated O_2_ concentrations. (A) qRT-PCR analyses of *REST* mRNA levels. (B) Immunoblot analysis of HPV18 E7 protein expression. HIF-1α, hypoxia marker; α-tubulin, loading control. (C, D) qRT-PCR analyses of HPV18 *E6/E7* (C) and *Rab3c* (D) mRNA expression. *Rab3c* served as a control gene which is repressed by REST (23). Graphs depict the mean expression levels relative to the results for shContr-1 under normoxia with standard deviations from 3 individual experiments (log2). Statistically significant differences (one-way ANOVA) from the respective shContr-1-treated condition are indicated by asterisks (***, *P* < 0.001; ns, not significant). Download FIG S1, TIF file, 0.9 MB. Copyright © 2019 Bossler et al. 2019 Bossler et al. This content is distributed under the terms of the Creative Commons Attribution 4.0 International license.

10.1128/mBio.02323-18.2 FIG S2 Mlx expression is not linked to E6/E7 repression under hypoxia. HeLa cells expressing shRNAs targeting Mlx (shMlx) or expressing control shRNA (shContr-1) were cultured for 24 h at the indicated O_2_ concentrations in medium containing 5.5 or 25 mM glucose. (A) qRT-PCR analyses of *Mlx* mRNA levels. (B) Immunoblot analysis of HPV18 E6/E7 and Mlx protein expression. HIF-1α, hypoxia marker. β-actin, loading control. (C, D) qRT-PCR analyses of HPV18 *E6/E7* (C) and *TXNIP* (D) mRNA expression. *TXNIP* served as a control gene, which is induced by 25 mM glucose in an Mlx-dependent manner (Carrie A. Stoltzman, Christopher W. Peterson, Kevin T. Breen, Deborah M. Muoio, Andrew N. Billin, Donald E. Ayer, Proc Natl Acad Sci USA 105:6912–6917, 2008, https://doi.org/10.1073/pnas.0712199105). Graphs depict the mean expression levels relative to the results for shContr-1 under normoxia (log2). Standard deviations of 3 individual experiments are indicated. Asterisks indicate statistically significant differences from the results for the respective shContr-1 as determined by one-way ANOVA (***, *P* < 0.001; *, *P* < 0.05; ns, not significant). Download FIG S2, TIF file, 2.0 MB. Copyright © 2019 Bossler et al. 2019 Bossler et al. This content is distributed under the terms of the Creative Commons Attribution 4.0 International license.

**FIG 1:**
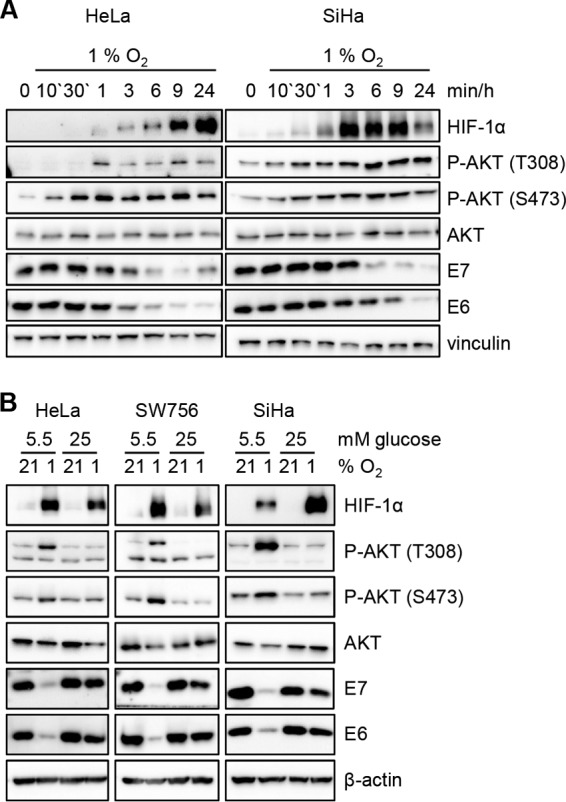
Hypoxia-induced AKT phosphorylation precedes and correlates with E6/E7 repression in a glucose-sensitive manner. (A) Time-course of hypoxia-induced AKT phosphorylation and E6/E7 repression. HeLa and SiHa cells were cultured for the indicated time periods under hypoxia, and protein expression of HIF-1α (hypoxia-linked marker), phosphorylated AKT (P-AKT T308 and P-AKT S473), pan-AKT (AKT), HPV16/-18 E6, and HPV16/-18 E7 was analyzed by immunoblotting (note that the phospho-AKT-specific antibodies recognize all three AKT isoforms, AKT1 to -3, when phosphorylated at corresponding sites, but for simplification, only the phosphorylation sites of AKT1 are indicated throughout the text). Vinculin, loading control. (B) Immunoblot analyses of HeLa, SW756, and SiHa cells cultured for 24 h under normoxia (21% O_2_) or hypoxia (1% O_2_) in medium containing 5.5 mM or 25 mM glucose. β-Actin, loading control.

Furthermore, we analyzed the PI3K/AKT pathway, since previous reports describe an upregulation of AKT activity in hypoxic cells (21, 22). This also holds true for HPV-positive cervical cancer cells, which show a clear induction of AKT phosphorylation at residues T308 and S473, starting as early as 30 min to 1 h after exposure to hypoxia (Fig. 1A). Phosphorylation of both of these residues is required for a complete activation of AKT (15). Downregulation of E6/E7 protein expression started at 3 to 9 h under hypoxia (Fig. 1A), thus occurring after induction of AKT activation. Notably, whereas hypoxia-induced AKT phosphorylation and E6/E7 repression were readily detected in HPV-positive cancer cells cultured in medium containing physiological serum glucose concentrations (5.5 mM), both responses were efficiently blocked by a high glucose supply (25 mM) (Fig. 1B). Hence, the activation of AKT precedes and correlates with E6/E7 repression in hypoxic cervical cancer cells in a glucose-sensitive manner, raising the possibility that AKT signaling is involved in hypoxic E6/E7 repression.

### Inhibitors of the PI3K/mTOR/AKT pathway reactivate hypoxic E6/E7 expression.

**FIG 2:**
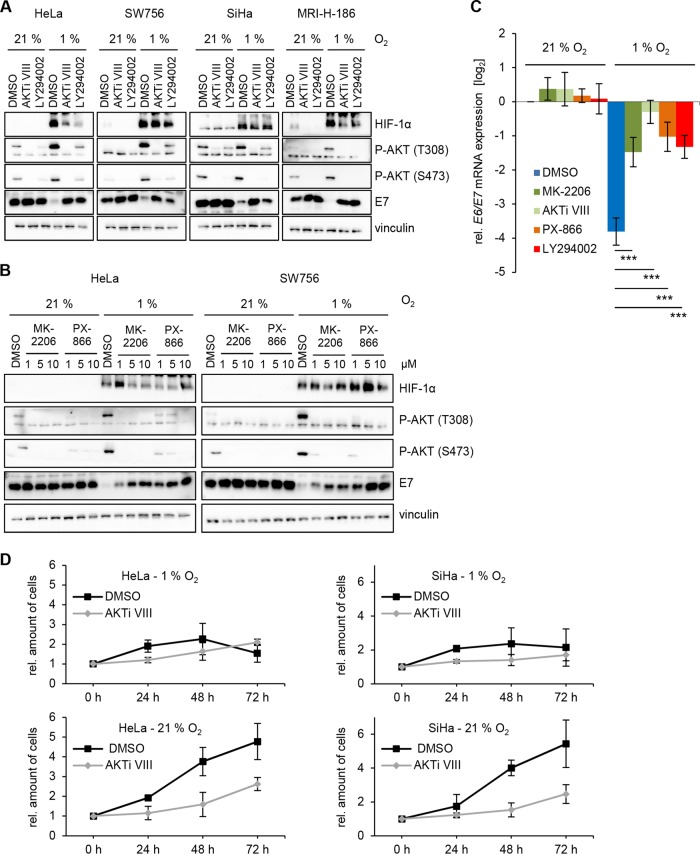
Inhibition of hypoxia-induced AKT phosphorylation counteracts E6/E7 repression. (A) HPV-positive HeLa, SW756, SiHa, and MRI-H-186 cervical cancer cells were treated with 10 µM AKTi VIII, 20 µM LY294002, or solvent control (DMSO) and incubated at the indicated O_2_ concentrations for 24 h. Immunoblot analyses of HIF-1α, phosphorylated AKT (P-AKT T308 and P-AKT S473), and HPV16/-18 E7 are shown. Vinculin, loading control. (B) HeLa and SW756 cells were treated with increasing concentrations of MK-2206 and PX-866 for 24 h and analyzed by immunoblotting. (C) qRT-PCR analyses of HPV18 *E6/E7* mRNA levels in HeLa cells treated with 3 µM MK-2206, 10 µM AKTi VIII, 3 µM PX-866, or 20 µM LY294002 for 24 h. Data shown are the mean expression levels under hypoxia relative to the expression levels in solvent (DMSO)-treated control cells under normoxia (log2). Standard deviations are depicted (*n* = 3). Asterisks indicate statistically significant differences as determined by one-way ANOVA (***, *P* < 0.001). (D) HeLa and SiHa cells were treated with 10 µM AKTi VIII and grown for the indicated time periods under hypoxia (top) or normoxia (bottom). Cell numbers relative to the time point 0 h after treatment were determined by quantitative crystal violet staining. Depicted are the mean values with standard deviations from 3 individual experiments.

To functionally analyze the role of AKT in hypoxic E6/E7 repression, HPV-positive cancer cells were treated with small molecule inhibitors targeting the PI3K/AKT pathway and then cultured under normoxia or hypoxia. The AKT-specific inhibitor AKTi VIII (26), as well as LY294002, an inhibitor of the upstream AKT activator PI3K (27), efficiently blocked phosphorylation of AKT (Fig. 2A). Under normoxia, no significant effects on E7 protein expression were detected upon treatment with the inhibitors. Importantly, however, inhibition of AKT phosphorylation under hypoxia efficiently counteracted E7 repression (Fig. 2A), indicating that the hypoxic downregulation of the HPV oncogenes is mediated through PI3K/AKT signaling.

LY294002, although commonly used to investigate PI3K signaling, can also affect the activity of mTOR (28) and has various other molecular targets (29, 30). To further specify the involvement of PI3K in hypoxic E6/E7 repression, we tested the highly selective class I PI3K inhibitor GDC-0941 (31), which also effectively interfered with hypoxic E7 repression (Fig. S3).

10.1128/mBio.02323-18.3 FIG S3 The PI3K inhibitor GDC-0941 counteracts hypoxic HPV oncogene repression. HeLa cells were treated with the indicated concentrations of GDC-0941 (or DMSO as solvent control) and incubated at the indicated O_2_ concentrations for 24 h. Immunoblot analyses of HIF-1α, phosphorylated AKT (P-AKT T308, P-AKT S473), and HPV18 E7 are shown. Vinculin, loading control. Download FIG S3, TIF file, 0.4 MB. Copyright © 2019 Bossler et al. 2019 Bossler et al. This content is distributed under the terms of the Creative Commons Attribution 4.0 International license.

The AKT- and glucose-dependent E6/E7 regulation under hypoxia is not restricted to cancer-derived HPV-positive cell lines but also observed in HPK II cells that were generated by immortalizing primary human keratinocytes with HPV16 DNA and which express *E6/E7* from the authentic viral promoter (Fig. S4) (32).

10.1128/mBio.02323-18.4 FIG S4 Hypoxic HPV oncogene repression is AKT and glucose dependent in HPV16 E6/E7-expressing human keratinocytes. HPK II cells were treated with 10 µM AKTi VIII or cultured with 25 mM glucose and incubated at the indicated O_2_ concentrations. Left, immunoblot analyses of phosphorylated AKT (P-AKT T308, P-AKT S473) and HPV16 E7. Vinculin, loading control. Right, qRT-PCR analyses of HPV16 *E6/E7* mRNA expression. Depicted are the mean expression levels under hypoxia relative to the results for solvent (DMSO)-treated control cells under normoxia (log2). Standard deviations (*n* = 4) are indicated. Asterisks below columns show statistically significant differences compared to the results for DMSO-treated cells under hypoxia as determined by one-way ANOVA (**, *P* < 0.01). Download FIG S4, TIF file, 0.5 MB. Copyright © 2019 Bossler et al. 2019 Bossler et al. This content is distributed under the terms of the Creative Commons Attribution 4.0 International license.

We extended our studies to two clinically tested compounds: the AKT-specific inhibitor MK-2206 (33) and the PI3K-specific inhibitor PX-866 (34). Both compounds also counteracted the hypoxic downregulation of E7 expression in a concentration-dependent manner (Fig. 2B). Interference with hypoxic E6/E7 repression was also detectable at the mRNA level for all tested PI3K/AKT inhibitors (Fig. 2C).

These findings raise the potentially clinically relevant question of whether the increased E6/E7 expression (a growth-promoting stimulus) under treatment with PI3K/AKT inhibitors may induce the proliferation of hypoxic HPV-positive cancer cells. However, HPV-positive cancer cells treated with AKTi VIII did not resume proliferation under hypoxia (Fig. 2D), despite the increase in E6/E7 expression (Fig. 2A and C). Moreover, treatment with AKTi VIII under normoxia inhibited the proliferation of HPV-positive cancer cells (Fig. 2D), albeit HPV oncogene expression was not downregulated (Fig. 2A and C).

**FIG 3:**
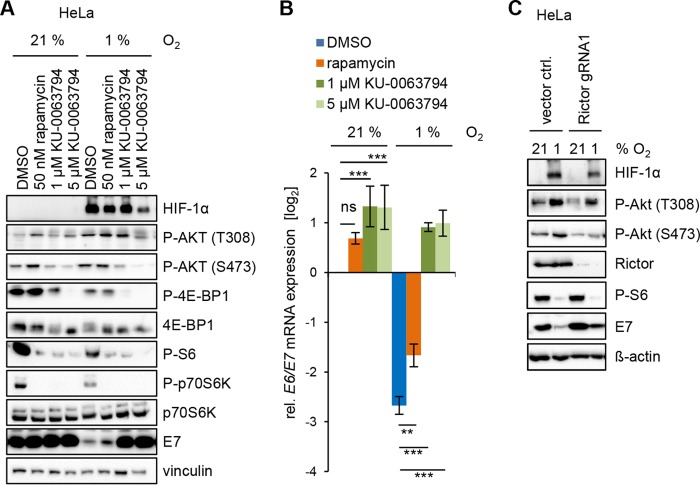
mTORC2 activity is required for repression of E6/E7 under hypoxia. (A) HeLa cells were treated with the indicated concentrations of rapamycin or KU-0063794, incubated for 1 h, and then cultured for an additional 23 h at the indicated O_2_ concentrations. The levels of HIF-1α, P-AKT T308, P-AKT S473, P-4E-BP1, 4E-BP1, P-S6, P-p70S6K, p70S6K, and HPV18 E7 were determined by immunoblotting. Vinculin, loading control. (B) Concomitant qRT-PCR analyses of HPV18 *E6/E7* mRNA levels in HeLa cells treated as described in the legend to panel A. Data shown are the mean expression levels relative to the expression levels in solvent (DMSO)-treated control cells under normoxia (log2). Standard deviations are depicted (*n* = 3). Asterisks indicate statistically significant differences as determined by one-way ANOVA (**, *P* < 0.01; ***, *P* < 0.001; ns, not significant). (C) Rictor expression was silenced in HeLa cells using CRISPR-Cas9 (Rictor gRNA1), and the cells cultured for 24 h at the indicated O_2_ concentrations. Control cells were transfected with the empty vector (vector ctrl). Immunoblot analyses show expression of HIF-1α, P-AKT T308, P-AKT S473, Rictor, P-S6, and HPV18 E6 and E7. β-Actin, loading control.

mTORC2 is the kinase responsible for phosphorylating AKT at S473 during canonical AKT signaling (35). Treatment with the mTOR inhibitor KU-0063794 prevented hypoxia-induced AKT phosphorylation at S473 and strongly counteracted the hypoxic repression of E7 (Fig. 3A). In contrast to KU-0063794, mTORC2 is largely insensitive to short-term treatment with rapamycin, an inhibitor of mTOR complex 1 (mTORC1) (36). Rapamycin treatment did not alter AKT activation or block E7 repression under hypoxia (Fig. 3A). Hypoxia-induced phosphorylation of AKT at T308 was only prevented by high concentrations of KU-0063794 (5 µM). The phosphorylation status of the mTORC1 targets 4E-BP1, p70S6K, and S6 showed the expected pattern after inhibitor treatment with phospho-4E-BP, only being blocked by KU-0063794 (37). These results were mirrored at the mRNA level. Rapamycin only weakly but KU-0063794 strongly counteracted the downregulation of *E6/E7* mRNA levels under hypoxia (Fig. 3B). A weak induction of *E6/E7* mRNA expression was detected under normoxia after treatment with the mTOR inhibitors. The functional role of mTORC2 in hypoxia-induced AKT activation and subsequent E6/E7 repression was further corroborated by clustered regularly interspaced short palindromic repeat (CRISPR)–CRISPR-associated protein 9 (Cas9)-mediated knockdown of the mTORC2 component Rictor (15), which also prevented increased hypoxic AKT phosphorylation at S473 and counteracted hypoxic E7 repression but did not alter the phosphorylation status of the mTORC1 downstream target S6 (Fig. 3C).

Collectively, these results indicate that AKT signaling mediates E6/E7 repression in hypoxic HPV-positive cancer cells, with PI3K and mTORC2 serving as upstream regulators of hypoxia-induced AKT activation.

### Hypoxic E6/E7 repression is mediated by AKT1 and AKT2.

The AKT isoforms, AKT1, -2, and -3, are each encoded by a different gene (15). Both AKT-specific inhibitors used in the present study (AKTi VIII and MK-2206) can target all three isoforms, with the highest affinity for AKT1 and the lowest for AKT3 (38, 39). Thus, we next aimed at investigating the contribution of the different AKT isoforms to hypoxic E6/E7 regulation.

AKT3 plays a major role in neuronal development and shows an enhanced activity in many melanomas (40, 41). We did not detect AKT3 expression in HeLa cells, in contrast to MeWo melanoma cells (Fig. S5). Thus, we focused our functional studies on the more ubiquitously expressed isoforms AKT1 and AKT2 (15).

10.1128/mBio.02323-18.5 FIG S5 AKT3 is not detected in HeLa cells. Immunoblot analysis of AKT3 expression in MeWo melanoma cells (lane 1) silenced by the CRISPR-Cas9 method with 2 different gRNAs (AKT3 gRNA1, AKT3 gRNA2) (lanes 2, 3), showing specificity of the signal for AKT3. In contrast to MeWo cells, AKT3 is undetectable in HeLa cells, under both normoxia and hypoxia (lanes 4, 5). β-Actin, loading control. Download FIG S5, TIF file, 0.5 MB. Copyright © 2019 Bossler et al. 2019 Bossler et al. This content is distributed under the terms of the Creative Commons Attribution 4.0 International license.

**FIG 4:**
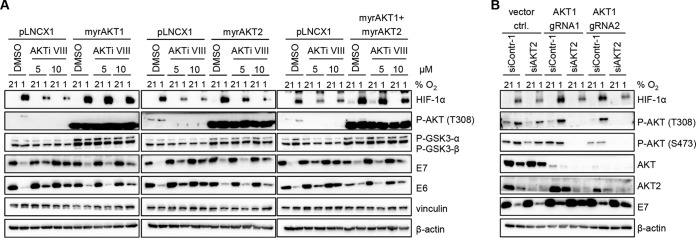
Repression of E6/E7 expression under hypoxia is mediated by AKT1 and AKT2. (A) HeLa cells transfected with control vector (pLNCX1) or expression vectors for constitutively active AKT1 and AKT2 (myrAKT1 and myrAKT2), alone or in combination, were treated with the indicated concentrations of AKTi VIII and cultured for 24 h at 21% or 1% O_2_. Immunoblot analyses of HIF-1α, P-AKT T308, P-GSK3-α/β, and HPV18 E6 and E7 are shown. Vinculin, β-actin, loading controls. (B) HeLa AKT1 knockdown single-cell clones (AKT1 gRNA1 and AKT1 gRNA4) of HeLa cells or controls containing the empty gRNA expression vector LentiCRISPRv1 (vector ctrl.) were transfected with siRNAs targeting AKT2 and cultured for 24 h at the indicated O_2_ concentrations. Protein expression of HIF-1α, P-AKT T308, P-AKT S473, pan-AKT (AKT), AKT2, and HPV18 E7 were analyzed by immunoblotting. β-Actin, loading control.

First, we tested whether increasing the activity of AKT1 or AKT2 could counteract the elevated E6/E7 expression induced by AKTi VIII treatment in hypoxic HPV-positive cancer cells. Hence, we overexpressed constitutively active myristoylated forms (42) of AKT1 and AKT2, alone or in combination, in HeLa cells (Fig. 4A). Ectopically expressed AKT can be distinguished from endogenous phosphorylated AKT as it has a lower molecular weight due to deletion of its pleckstrin homology (PH) domain (43). Functionality of the constitutively active AKT proteins was verified by detecting an increase in phosphorylation of glycogen synthase kinase 3-α and -β (GSK3-α/β) (Fig. 4A), a well-characterized AKT target (44). Overexpression of constitutively active AKT1, AKT2, or AKT1 and AKT2 together did not alter E6/E7 expression under normoxia. However, the AKTi VIII-mediated increase in E6/E7 expression under hypoxia was clearly diminished upon overexpression of active AKT1 and AKT2, alone or in combination (Fig. 4A), indicating functional redundancy of AKT1 and AKT2.

To further explore this issue, AKT1 expression was silenced in HeLa cells by the CRISPR-Cas9 method. Different guide RNAs (gRNAs) targeting AKT1 were used, and single-cell clone selection was performed. Two different single-cell clones were transiently transfected with small interfering RNAs (siRNAs) targeting AKT2 and cultured under normoxia or hypoxia. Immunoblot analyses revealed that despite an efficient reduction in total AKT levels, AKT1 knockdown alone did not deplete phosphorylated AKT (Fig. 4B). Silencing of AKT2 expression by siRNA in control single-cell clones did not significantly alter AKT phosphorylation. Notably, however, the combined silencing of AKT1 and AKT2 efficiently depleted phosphorylated AKT (Fig. 4B). Moreover, whereas a weak increase in E7 levels was observed upon single knockdown of AKT1, hypoxic E7 repression was more efficiently counteracted upon silencing the expression of both AKT isoforms. Taken together, these results indicate that AKT1 and AKT2 both mediate hypoxic E6/E7 repression and act in a functionally redundant manner, while arguing against the involvement of AKT3.

### Hypoxic repression of E6/E7 occurs at the transcriptional level.

**FIG 5:**
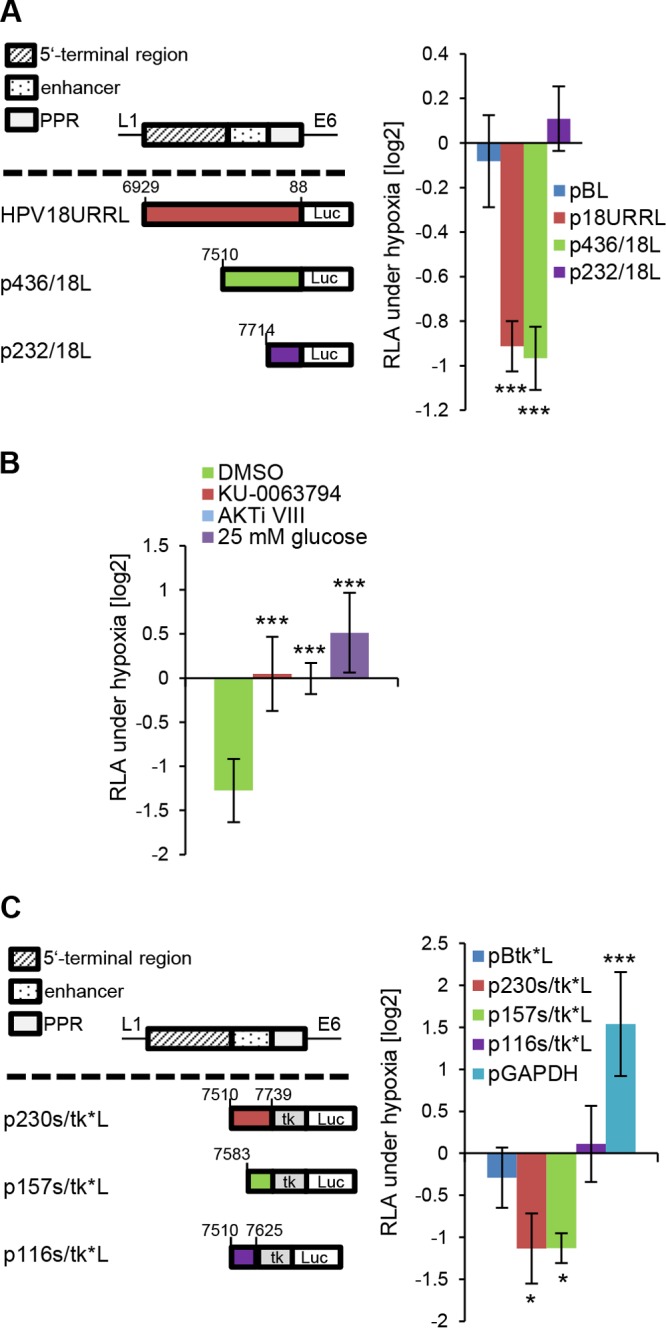
Regulation of *E6/E7* transcription under hypoxia. (A) Top left, schematic presentation of the 825-bp HPV18 URR with the central 230-bp enhancer and the promoter proximal region (PPR) containing the *E6/E7* promoter at the 3′ terminus (45). Bottom left, luciferase reporter constructs containing the firefly luciferase gene under the control of the complete HPV18 URR (p18URRL) or deletion constructs thereof (p436/18L and p232/18L). Nucleotide positions are according to reference 76. pBL, basic luciferase plasmid (45). Right, luciferase reporter constructs were analyzed in HeLa cells cultured for 24 h under hypoxia or normoxia. Shown are the relative luciferase activities (RLA) of the individual reporter plasmids under hypoxia compared to the RLA under normoxia (log2). Standard deviations are depicted (*n* = 4). Asterisks above bars show statistically significant differences from the results for pBL as determined by one-way ANOVA (***, *P* < 0.001). (B) Effects of 1 µM KU-00637794, 10 µM AKTi VIII, and 25 mM glucose on the hypoxic repression of p436/18L. Shown are the RLA under hypoxia compared to the RLA of solvent (DMSO)-treated control cells under normoxia (log2). Standard deviations are depicted (*n* = 5). Asterisks above columns show statistically significant differences compared to the results for DMSO-treated cells as determined by one-way ANOVA (***, *P* < 0.001). (C) Reporter assays (*n* = 5) of the HPV18 enhancer (p230s/tk*L) or deletion constructs thereof (p157s/tk*L and p116s/tk*L) upstream from the HSV *TK* promoter. pBtk*L, control vector devoid of HPV enhancer sequences (45). Shown are the RLA of the individual reporter plasmids under hypoxia compared to the RLA under normoxia (log2). Asterisks above columns show statistically significant differences compared to the results for pBtk*L as determined by one-way ANOVA (*, *P* < 0.05; ***, *P* < 0.001). pGAPDH, positive control.

Since hypoxia-induced E6/E7 repression is detectable at the mRNA level (11), we investigated whether hypoxia affects the activity of the HPV18 *E6/E7* transcriptional promoter. The activity of a reporter construct containing the *E6/E7* promoter in the context of the complete 825-bp HPV18 transcriptional control region (upstream regulatory region [URR]) (p18URRL) (45) was significantly reduced under hypoxia (Fig. 5A). This was also observed for a reporter construct which only contains the 230-bp transcriptional enhancer linked to the promoter-proximal region (PPR), after deletion of the 5′-terminal 389-bp portion of the HPV18 URR (p436/18L). The hypoxic repression of the *E6/E7* promoter was counteracted by inhibiting AKT (AKTi VIII) or mTOR (KU-00637794) signaling, as well as by excess glucose (25 mM) (Fig. 5B). The activity of a further 5′-terminally truncated construct which only encompasses the PPR (p232/18L) was not reduced under hypoxia (Fig. 5A), indicating that the HPV enhancer plays a critical role for hypoxic E6/E7 repression. The homologous HPV18 PPR appears to be dispensable for this effect, since a construct linking the 230-bp HPV18 enhancer to the heterologous HSV *TK* (thymidine kinase) promoter (p230s/tk*L) was also significantly repressed under hypoxia (Fig. 5C). Further 5′- and 3′-terminal deletions within the HPV18 enhancer showed that a 3′-terminal 157-bp fragment (p157s/tk*L) was sufficient to confer hypoxia-mediated repression, but a 116-bp 5′-terminal fragment (p116s/tk*L) was not (Fig. 5C). Glyceraldehyde-3-phosphate dehydrogenase gene (*GAPDH*) promoter activity was monitored as a positive control and showed the expected stimulation under hypoxia (Fig. 5C) (46).

Since these findings indicate that the repressive effect of hypoxia involves transcriptional regulation, we also studied potential alterations of the epigenetic status of the HPV *E6/E7* promoter under hypoxia using SiHa cells, which contain only one or two HPV16 integrates (47). We assessed the methylation of the viral DNA via methylated DNA immunoprecipitation (MeDIP) (48) and found that methylation levels at the HPV16 URR remained low under hypoxia, suggesting that DNA methylation is not involved in silencing of E6/E7 expression (Fig. S6A). In line with a previous report (49), the *L1* and *L2* genes generally exhibited higher DNA methylation levels than the URR (Fig. S6A). Next, using chromatin immunoprecipitation (ChIP), we analyzed trimethylation of histone H3 at lysine 4 (H3K4me3) and trimethylation of histone H3 at lysine 27 (H3K27me3), which are linked to active and inactive genes, respectively (50). Enrichment of both H3K4me3 and H3K27me3 was detected at all regions investigated, including the HPV16 URR (Fig. S6B). This is in line with a global increase in H3K4me3 and H3K27me3 levels observed in SiHa and HeLa cells under hypoxia (Fig. S6C). Notably, H3K4me3 levels at the transcriptionally silenced *L1* and *L2* genes remained low, whereas H3K27me3 levels were generally higher at *L1/L2* than at the URR (Fig. S6B). Hence, hypoxic repression of E6/E7 was not associated with an increase in DNA methylation but was linked to alterations in H3K4me3 and H3K27me3 occupancy at the viral URR.

10.1128/mBio.02323-18.6 FIG S6 Epigenetic analyses of the HPV16 URR under hypoxia. (A) Depicted are real-time qPCR analyses following MeDIP of SiHa cells incubated for 24 h at the indicated O_2_ concentrations. Primers for two regions in the HPV16 URR (HPV16URR_1, HPV16URR_2) and for one region in the HPV16 *L2* and *L1* gene (HPV16L1, HPV16L2) were used. Tuba1C = negative control, unmethylated. CpG 4, positive control, methylated. Shown are the mean percentages of input from 3 independent experiments. Standard deviations are indicated. (B) SiHa cells were incubated for 24 h at the indicated O_2_ concentrations, and ChIP using antibody against H3K27me3 (left) or H3K4me3 (right) was performed, followed by real-time qPCR analyses. Primers for HPV16 were applied as described for panel A. C1orf43, H3K4me3 positive control; HOXC13, H3K27me3 positive control. (C) Left, hypoxia increases total H3K27me3 and H3K4me3 amounts in HeLa and SiHa cells. Cells were cultured for 24 h at the indicated O_2_ concentrations, and HIF-1α, H3K27me3, H3K4me3 and HPV16/18 E7 protein expression analyzed by immunoblotting. β-Actin, loading control. Right, hypoxia-linked increases in total H3K27me3 and H3K4me3 levels are counteracted by inhibition of AKT or PI3K signaling. SiHa cells were treated with 10 µM AKTi VIII or 20 µM LY294002 and cultured for 24 h at the indicated O_2_ concentrations. Immunoblots of HIF-1α, phosphorylated AKT (P-AKT T308, P-AKT S473), H3K27me3, H3K4me3, and HPV16 E7 are shown. β-Actin, loading control. Download FIG S6, TIF file, 1.7 MB. Copyright © 2019 Bossler et al. 2019 Bossler et al. This content is distributed under the terms of the Creative Commons Attribution 4.0 International license.

Taken together, these data indicate that hypoxic repression of HPV18 E6/E7 occurs, at least in part, at the transcriptional level and involves the 157-bp 3′-terminal portion of the viral enhancer.

### Proteome analysis of hypoxic HPV-positive cancer cells.

**FIG 6:**
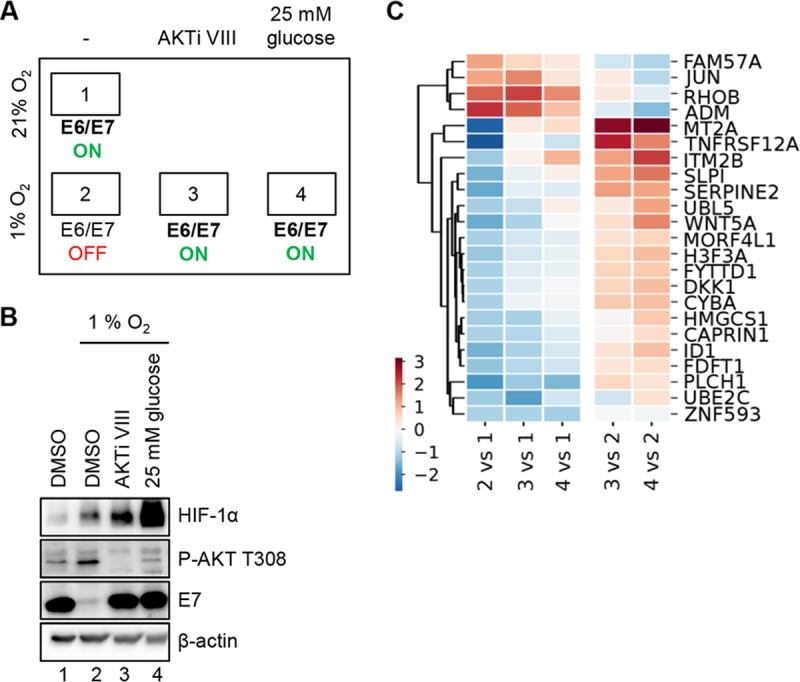
Proteome analyses of proteins differentially expressed under normoxia and hypoxia. TMT-mass spectrometry analyses of SiHa cells cultured under normoxia and hypoxia and under hypoxia in the presence of 10 µM AKTi VIII or 25 mM glucose. (A) Schematic illustration of the four treatment conditions (conditions 1 to 4) and the corresponding regulation of E6/E7 expression. (B) Accompanying immunoblot showing protein expression of HIF-1α, P-AKT T308, and HPV16 E7 under the four treatment conditions (numbered as in panel A) used for proteome analyses. β-Actin, loading control. (C) Heat map (hierarchical clustering) depicting relative protein expression levels (log2 fold change), filtered for differentially expressed proteins (FDR < 0.05) under hypoxia (condition 2) compared to normoxia (condition 1) with a log2 fold change of ≥+1 or ≤−1. Upregulation is shown in red, downregulation in blue (see color scheme at the lower left). The left three columns reflect protein expression under the three different hypoxic conditions analyzed (untreated [condition 2] or in the presence of AKTi VIII [condition 3] or 25 mM glucose [condition 4]), relative to untreated control cells under normoxia (condition 1). The right two columns show comparisons of hypoxic cells treated with AKTi VIII (condition 3) or high glucose (condition 4) relative to untreated hypoxic cells (condition 2).

Cornerstones of the regulatory phenomena observed in hypoxic HPV-positive cancer cells are as follows: (i) hypoxia blocks E6/E7, an effect that can be counteracted by (ii) AKT inhibition and by (iii) high glucose supply. To gain further insights into the underlying regulatory circuits, mass spectrometry-based quantitative proteome analyses were performed comparing normoxic and hypoxic SiHa cells and assessing their response toward AKTi VIII and 25 mM glucose under hypoxia (Fig. 6A and B). These experiments should also provide information about cellular proteins and pathways that are regulated under these different experimental conditions in parallel or inversely with HPV E6/E7. These pathways could be subject to the same regulatory principles as E6/E7 or, alternatively, could themselves act as upstream regulators or downstream effectors of E6/E7 in hypoxic HPV-positive cancer cells.

We detected 46 proteins (among a total of 5,966 measured proteins) which were differentially expressed (false discovery rate [FDR] < 0.05) under hypoxic conditions. Among those, 23 proteins showed ≥2-fold (log2 fold change of ≥+1 or ≤−1) up- or downregulation (4 upregulated and 19 downregulated). The heat map in Fig. 6C illustrates changes in the expression of these 23 proteins under hypoxia compared to their expression under normoxia (Fig. 6C, left columns), as well as their regulation in hypoxic cells treated with AKTi VIII or high glucose concentrations (25 mM) compared to that in untreated hypoxic cells (Fig. 6C, right columns; also Table S1). We chose 5 of these proteins (DKK1, ITM2B, TNFRSF12A, SLPI, and Wnt5a) for further validation by immunoblot analysis. All factors exhibit concordant changes under the different treatment conditions as observed in the proteome analysis (Fig. S7A). Furthermore, we tested their expression in HeLa cells. Whereas DKK1 protein is undetectable, all four of the other factors show the same response pattern in HeLa cells as in SiHa cells (Fig. S7B).

10.1128/mBio.02323-18.7 FIG S7 Validation of selected hits of the proteome analyses. (A) SiHa cells were cultured under normoxia and hypoxia and under hypoxia in the presence of 10 µM AKTi VIII or 25 mM glucose. Left, immunoblot analyses of phosphorylated AKT (P-AKT T308, P-AKT S473), HPV16 E7, Wnt5a/b, SLPI, TNFRSF12A, ITM2B, and DKK1. HIF-1α, hypoxia marker; β-actin, vinculin, loading controls. Right, qRT-PCR analyses for HPV16 *E6/E7*, *DKK1*, *Wnt5a*, *SLPI*, and *ITM2B*. Depicted are the mean expression levels under hypoxia relative to the results for solvent (DMSO)-treated control cells under normoxia (log2). Standard deviations (*n* = 3) are indicated. Asterisks below or above columns show statistically significant differences compared to the results for DMSO-treated cells under hypoxia as determined by one-way ANOVA (***, *P* < 0.001; **, *P* < 0.01; *, *P* < 0.05). (B) Immunoblot analyses of HeLa cells treated as described for SiHa cells in panel A. Protein expression of phosphorylated AKT (P-AKT T308, P-AKT S473), HPV18 E7, Wnt5a/b, SLPI, TNFRSF12A; and ITM2B is shown. HIF-1α, hypoxia marker; β-actin, vinculin, loading controls; n.d., not detectable. Download FIG S7, TIF file, 2.0 MB. Copyright © 2019 Bossler et al. 2019 Bossler et al. This content is distributed under the terms of the Creative Commons Attribution 4.0 International license.

10.1128/mBio.02323-18.8 TABLE S1 Log2-fold changes (log2FC) and adjusted *P* values (adj. p-value) of proteins detected. Download Table S1, XLSX file, 1.3 MB. Copyright © 2019 Bossler et al. 2019 Bossler et al. This content is distributed under the terms of the Creative Commons Attribution 4.0 International license.

Collectively, the proteome data indicate that the regulation of several proteins is partially or fully reverted when hypoxic cells are treated with AKTi VIII or high glucose. These proteins include factors that are directly linked to the PI3K/AKT signaling pathway, such as adrenomedullin (ADM) (51), FAM57A (CT120) (52), or metallothionein 2A (MT2A) (53). Furthermore, two inhibitors of canonical Wnt signaling, Wnt5a (54) and DKK1 (55), were significantly downregulated under hypoxia in an AKT- and glucose-dependent manner, providing a possible link between AKT and the canonical Wnt signaling pathway under hypoxia.

## DISCUSSION

Hypoxic HPV-positive cancer cells can strongly downregulate HPV E6/E7 oncogene expression, entering a dormant state, which could be associated with decreased therapeutic susceptibility to chemotherapy, immunotherapy, and prospective E6/E7 inhibitors (11). This work aimed to gain insights into the underlying mechanism and reveals that the downregulation of E6/E7 is mediated by the hypoxic activation of the PI3K/AKT signaling cascade. This conclusion is supported by the findings that (i) hypoxia-induced phosphorylation of AKT precedes downregulation of E6/E7 and, like hypoxic E6/E7 repression, is sensitive to high glucose concentrations, (ii) chemical inhibitors targeting the PI3K/AKT pathway block hypoxic E6/E7 repression, an effect that is counteracted by increasing AKT signaling in HPV-positive cancer cells, and (iii) knockdown experiments identify the AKT1 and AKT2 isoforms as being crucial for hypoxic E6/E7 repression.

Although individual AKT isoforms can exert specific activities, as indicated by the different phenotypes of isoform-specific knockout mice (56, 57), our results indicate functional redundancy of AKT1 and AKT2 during hypoxic E6/E7 repression. The ectopic expression of either constitutively active AKT1 or AKT2 can counteract the elevation of E6/E7 levels that is induced by AKTi VIII treatment under hypoxia, and the concomitant ectopic expression of both constitutively active AKT isoforms did not further augment this effect. Moreover, only the combined knockdown of both isoforms depleted the cell of phosphorylated AKT and efficiently counteracted E7 repression under hypoxia. Functional redundancy of AKT1 and AKT2 in hypoxic repression of E6/E7 is in accordance with many overlapping functions of AKT1 and AKT2 and with their ability to, at least partially, compensate for each other, e.g., as indicated by the lethality of knockout mice lacking both AKT1 and AKT2, in contrast to viable single-isoform knockout mice (15, 58).

Targeted inhibition of the PI3K/AKT pathway is currently being investigated as a possible new strategy for cancer therapy in the clinic (16). The observation in this study that PI3K/AKT inhibitors are linked to increased viral oncogene expression in hypoxic HPV-positive cancer cells may therefore raise concern, since E6/E7 expression is oncogenic and represents a strong proproliferative stimulus (1, 7). However, despite the increased E6/E7 expression levels in hypoxic cervical cancer cells following AKT inhibition, we found that the cells did not resume proliferation. Moreover, AKT inhibition efficiently blocks the proliferation of normoxic HPV-positive cancer cells even though these cells maintain their E6/E7 expression. Collectively, these results indicate that the antiproliferative effect of AKT inhibition is dominant over the growth-promoting potential of E6/E7 expression, both under hypoxic and normoxic conditions.

Surprisingly, although hypoxia poses an important determinant for the malignant growth and the therapeutic resistance of tumors (12–14) and the AKT signal cascade is recognized to play a central role for many cancer entities (15), data on the mechanisms underlying AKT activation under hypoxia are still sparse. Our findings show that the phosphorylation of AKT and subsequent E6/E7 repression under hypoxia depend on active PI3K and mTORC2 as upstream regulators, both representing key players of the canonical pathway of AKT activation (15). Interestingly, it has recently been reported that AKT can be inhibited under normoxia in a hypoxia-inducible factor (HIF)-independent manner via the O_2_-dependent hydroxylase EgIN1, resulting in enhanced AKT activation when O_2_ is lacking (59). Whether impaired O_2_-dependent hydroxylation of AKT also plays a role in AKT activation and E6/E7 repression in hypoxic HPV-positive cancer cells remains to be elucidated.

Moreover, our findings link the PI3K/mTORC2/AKT signaling cascade to the regulation of the HPV oncogenes. In line with the potential of the AKT pathway to alter transcription (15), our results indicate that E6/E7 repression under hypoxia is linked to transcriptional repression of the *E6/E7* promoter. While we did not observe a connection between DNA methylation and hypoxic E6/E7 repression, we detected increases of both the repressive histone marker H3K27me3 and the activating histone marker H3K4me3 at the HPV16 URR. This was associated with a global increase of H3K27me3 and H3K4me3 levels, which could be attributable to reduced activity of O_2_-dependent histone demethylases of the JARID1 family and JMJD3 (60–62). Enrichment of H3K27me3 can correlate with transcriptional repression even in the presence of H3K4me3 under certain conditions (62), and thereby may contribute to E6/E7 downregulation under hypoxia. In addition, distinct *cis*-regulatory elements of the viral URR appear to be involved in hypoxia-linked E6/E7 repression, since we found that a 157-bp 3′-terminal fragment of the HPV18 enhancer is sufficient to mediate transcriptional repression under hypoxia. A detailed analysis of the hypoxic regulation of transcription factors binding to this region, which, among others, include AP1 family members, Oct1, NF1, C/EBP proteins, and YY1 (45, 63), and their possible connection to AKT signaling in hypoxic HPV-positive cancer cells could provide additional mechanistic insights.

**FIG 7:**
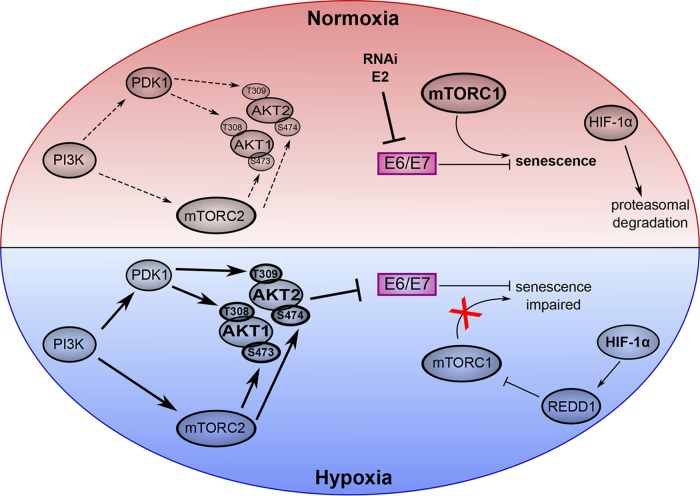
Cross talk between oncogenic HPVs and the PI3K/mTOR/AKT signaling cascade. Top, normoxia. Top right, experimental repression of E6/E7 (e.g., by RNA interference [RNAi] or by the viral transrepressor E2) leads to rapid senescence of HPV-positive cancer cells (8–10). The efficiency of senescence induction is dependent on intact mTORC1 signaling (11). HIF-1α is unstable under normoxia. Top left, canonical PI3K/mTORC2/AKT signaling. PI3K via PDK1 (phosphoinositide dependent kinase-1) and mTORC2 activates AKT signaling through mediating the phosphorylation of AKT1 at amino acids T308 and S473 (and of T309 and S474 for AKT2) (15). Bottom, hypoxia. Bottom right, HIF-1α is stabilized and stimulates mTORC1-inhibitory REDD1 expression (77). The resulting interference with mTORC1 signaling leads to impaired senescence (11). Bottom left, E6/E7 repression in hypoxic HPV-positive cancer cells depends on the hypoxia-induced increase of AKT1 and AKT2 phosphorylation. This regulation requires the function of the canonical upstream AKT activators PI3K and mTORC2.

Collectively, the findings of this study underline the significance of PI3K/AKT/mTOR signaling for the virus-host cell cross talk in HPV-positive cancer cells (Fig. 7). We previously observed that efficient induction of senescence, which occurs under normoxia upon E6/E7 repression (8–10), is rapamycin sensitive and requires active mTORC1 signaling (11). Under hypoxia, however, mTORC1 signaling is impaired, allowing HPV-positive cells to evade senescence despite E6/E7 repression (11). In the present work, we show that the regulation of viral oncogene repression under hypoxia is also connected to the PI3K/AKT/mTOR regulatory circuit, in this case being rapamycin insensitive and involving the mTORC2 complex (Fig. 7), as further corroborated by silencing RICTOR expression. In this cascade, both PI3K and mTORC2 are required as upstream regulators for the hypoxia-induced activation of AKT1 and AKT2, which mediate the repression of the viral oncogenes in hypoxic HPV-positive cancer cells.

It is important to note that the modulation of AKT signaling affected viral oncogene expression only in hypoxic and not in normoxic HPV-positive cancer cells. As a basis for future studies of this complex regulation, we also performed comparative proteome analyses of normoxic and hypoxic cells and hypoxic cells treated with an AKT inhibitor or with high glucose concentrations, the latter two treatments counteracting hypoxic E6/E7 repression. Conceivably, proteins that are regulated in parallel or inversely with E6/E7 under these different experimental conditions could represent (i) factors which are not linked to E6/E7 repression but also underlie AKT-dependent modulation under hypoxia, (ii) factors acting downstream from AKT and upstream from E6/E7 during hypoxic E6/E7 repression, or (iii) factors acting downstream from E6/E7 and which are affected by the downregulation of the viral oncogenes in hypoxic cells. The limited number of proteins fulfilling these criteria included Wnt5a and DKK1, which were downregulated under hypoxia, an effect that was counteracted by AKTi VIII and high glucose treatment. Interestingly, both proteins can act as negative regulators of Wnt/β-catenin signaling (54, 55) and thereby may not only be involved in E6/E7 repression but could also connect AKT and Wnt signaling in hypoxic cells. We also identified proteins whose expression is altered under hypoxia but not reverted by treatment with AKTi VIII or high glucose. These factors could comprise upstream regulators of hypoxic AKT activation. Interestingly, these include RhoB and c-Jun, both being upregulated under hypoxia and both having the potential to stimulate AKT signaling, at least under normoxic conditions (64–66). Future studies of these differentially regulated factors are warranted to assess their functional connection to the hypoxic response of the PI3K/mTORC2/AKT signaling cascade in general, as well as their contribution to the repression of the E6/E7 oncogenes in hypoxic HPV-positive cancer cells.

## MATERIALS AND METHODS

### Cell culture, treatments, and transfections.

HPV18-positive HeLa and SW756 and HPV16-positive SiHa and MRI-H-186 cervical carcinoma cells and MeWo melanoma cells were obtained from the tumor bank of the German Cancer Research Center (DKFZ) or from the American Tissue Culture Collection. HPK II cells were characterized in reference 32. Cells were maintained in Dulbecco modified Eagle medium (DMEM) or RPMI (MRI-H-186) medium supplemented with 10% fetal calf serum (FCS; Life Technologies), 2 mM l-glutamine, 100 U/ml penicillin, and 100 µg/ml streptomycin (Sigma-Aldrich). Standard cell culture medium had a glucose concentration of 5.5 mM. Cells were either cultured under normoxia (21% O_2_, 5% CO_2_) in a humidified incubator or under hypoxia (1% O_2_, 5% CO_2_) in the InvivO_2_ 400 physiological oxygen workstation (Ruskinn Technology Ltd., Bridgend, United Kingdom).

The following PI3K/AKT/mTOR inhibitors, diluted in dimethyl sulfoxide (DMSO), were used for treatment: KU-0063794, AKTi VIII (Sigma-Aldrich), LY294002 (Cayman Chemical), rapamycin, MK-2206 (AdipoGen), PX-866 (Focus Biomolecules), and GDC-0941 (Selleck Chemicals).

All plasmids were transfected by calcium phosphate coprecipitation (67). To generate knockdown cells by the CRISPR-Cas9 technique, transfected cells were selected for up to 5 days using puromycin (1 µg/ml). Cells were then cultured in standard medium and either used directly as a pool for further experiments (Fig. 3C; also Fig. S4 in the supplemental material) or split for generating single-cell clones (Fig. 4B). Control cells were transfected with the empty vector containing no guide RNA (gRNA) sequence. Synthetic siRNAs were transfected using DharmaFECT I (Thermo Fisher Scientific) according to the manufacturer’s instructions, with a final siRNA concentration of 20 nM.

### Plasmids and siRNAs.

Synthetic siRNAs were purchased from Life Technologies. shRNAs were expressed from pSuper (68). The si-/shRNA target sequences were as follows: shREST-1, 5′-GCUUAUUAUGCUGGCAAAU-3′; shREST-2, 5′-GCUGCGGCUACAAUACUAA-3′; shREST-3, 5′-GUGACUACCAGAACUCGAA-3′; shMlx-1, 5′-UCAUGAAAGUGAACUAUGA-3′; shMlx-2, 5′-CUGACCAGGUCAAGUUCAA-3′; siAKT2-1, 5′-UGACUUCGACUAUCUCAAA-3′; siAKT2-2, 5′-CAACUUCUCCGUAGCAGAA-3′; and si-/shContr-1, 5′-CAGUCGCGUUUGCGACUGG-3′ (contains at least four mismatches to all known human genes). si-/shRNAs were pooled at equimolar concentrations to minimize off-target effects. gRNAs against AKT1 and AKT3 were expressed from vector LentiCRISPRv1, and gRNAs against Rictor were expressed from LentiCRISPRv2 (kind gifts from Feng Zhang; Addgene plasmids no. 49535 and no. 52961) (69). The following gRNA target sequences were used: AKT1 gRNA1, 5′-GACGTGGCTATTGTGAAGGA-3′; AKT1 gRNA2, 5′-TGTCATGGAGTACGCCAACG-3′; AKT3 gRNA1, 5′-GAGAATATATAAAAAACTGG-3′; AKT3 gRNA2, 5′-GCCACTGAAAAGTTGTTGAG-3′; and Rictor gRNA1, 5′-TGTCTTCACATGCTTCATCG-3′. Expression vectors for constitutively active AKT1 (myrAKT1) and AKT2 (myrAKT2) were obtained from Addgene (plasmids no. 15989 and no. 27294, respectively; kind gifts from Morris Birnbaum [70]). Luciferase reporter plasmids for the HPV18 URR (45) and pGAPDH (71) were described previously.

### Luciferase reporter assays.

For luciferase assays, cells were seeded in 6-cm dishes and transfected with 3 µg reporter plasmid or basic vector. To adjust for variations in transfection efficiency, 0.2 µg pCMV-β-galactosidase was included as an internal standard. Cell lysis and measurement of luciferase activity was carried out 48 h after transfection as previously described (72). Experiments were performed independently at least 4 times in duplicates, and data are presented as fold changes relative to the results under hypoxia, after logarithmic transformation. Statistical significance was determined by one-way analysis of variance (ANOVA) (*, *P* < 0.05; ***, *P* < 0.001).

### Immunoblot and qRT-PCR analyses.

Protein extraction, immunoblotting, RNA extraction, and quantitative reverse-transcription PCR (qRT-PCR) were performed as described previously (73) and detailed in Text S1 in the supplemental material.

10.1128/mBio.02323-18.9 TEXT S1 Supplemental methods. Download Text S1, DOCX file, 0.04 MB. Copyright © 2019 Bossler et al. 2019 Bossler et al. This content is distributed under the terms of the Creative Commons Attribution 4.0 International license.

### Determination of cell growth.

Cell numbers were quantitated by staining with crystal violet (74). Experiments were performed at least thrice in quadruplicates. Cells were seeded in 96-well plates and stained with 30 µl formaldehyde-crystal violet (12 mM crystal violet, 29 mM NaCl, 3.7% formaldehyde, 22% ethanol) 0, 24, 48, and 72 h after treatment. Plates were washed and dried. For quantification of cells, 30 µl 33% acetic acid was added to dissolve cell-bound crystal violet. Absorbance was measured at 570 nm. Data are presented relative to the time point 0 h after treatment.

### TMT-mass spectrometry analyses.

Quantitative proteome analyses were performed by tandem mass tag (TMT)-mass spectrometry (75), as detailed in Text S1.

## References

[1] Zur Hausen H 2002 Papillomaviruses and cancer: from basic studies to clinical application. Nat Rev Cancer 2:342–350. doi:10.1038/nrc798. 12044010

[2] de Martel C, Plummer M, Vignat J, Franceschi S 2017 Worldwide burden of cancer attributable to HPV by site, country and HPV type. Int J Cancer 141:664–670. doi:10.1002/ijc.30716. 28369882 PMC5520228

[3] Schiller J, Lowy D 2018 Explanations for the high potency of HPV prophylactic vaccines. Vaccine 36:4768–4773. doi:10.1016/j.vaccine.2017.12.079. 29325819 PMC6035892

[4] Hellner K, Munger K 2011 Human papillomaviruses as therapeutic targets in human cancer. J Clin Oncol 29:1785–1794. doi:10.1200/JCO.2010.28.2186. 21220591 PMC3675666

[5] Hoppe-Seyler K, Bossler F, Braun JA, Herrmann AL, Hoppe-Seyler F 2018 The HPV E6/E7 oncogenes: key factors for viral carcinogenesis and therapeutic targets. Trends Microbiol 26:158–168. doi:10.1016/j.tim.2017.07.007. 28823569

[6] Bruni L, Diaz M, Barrionuevo-Rosas L, Herrero R, Bray F, Bosch FX, de Sanjosé S, Castellsagué X 2016 Global estimates of human papillomavirus vaccination coverage by region and income level: a pooled analysis. Lancet Glob Health 4:e453–e463. doi:10.1016/S2214-109X(16)30099-7. 27340003

[7] McLaughlin-Drubin ME, Münger K 2009 Oncogenic activities of human papillomaviruses. Virus Res 143:195–208. doi:10.1016/j.virusres.2009.06.008. 19540281 PMC2730997

[8] Goodwin EC, Yang E, Lee CJ, Lee HW, DiMaio D, Hwang ES 2000 Rapid induction of senescence in human cervical carcinoma cells. Proc Natl Acad Sci U S A 97:10978–10983. 11005870 10.1073/pnas.97.20.10978 PMC27134

[9] Wells SI, Francis DA, Karpova AY, Dowhanick JJ, Benson JD, Howley PM 2000 Papillomavirus E2 induces senescence in HPV-positive cells via pRB- and p21(CIP)-dependent pathways. EMBO J 19:5762–5771. doi:10.1093/emboj/19.21.5762. 11060027 PMC305788

[10] Magaldi TG, Almstead LL, Bellone S, Prevatt EG, Santin AD, DiMaio D 2012 Primary human cervical carcinoma cells require human papillomavirus E6 and E7 expression for ongoing proliferation. Virology 422:114–124. doi:10.1016/j.virol.2011.10.012. 22056390 PMC3229657

[11] Hoppe-Seyler K, Bossler F, Lohrey C, Bulkescher J, Rösl F, Jansen L, Mayer A, Vaupel P, Dürst M, Hoppe-Seyler F 2017 Induction of dormancy in hypoxic human papillomavirus-positive cancer cells. Proc Natl Acad Sci U S A 114:E990–E998. doi:10.1073/pnas.1615758114. 28115701 PMC5307428

[12] Vaupel P, Mayer A 2007 Hypoxia in cancer: significance and impact on clinical outcome. Cancer Metastasis Rev 26:225–239. doi:10.1007/s10555-007-9055-1. 17440684

[13] Gadducci A, Guerrieri ME, Greco C 2013 Tissue biomarkers as prognostic variables of cervical cancer. Crit Rev Oncol Hematol 86:104–129. doi:10.1016/j.critrevonc.2012.09.003. 23031678

[14] Hoppe-Seyler K, Mandl J, Adrian S, Kuhn BJ, Hoppe-Seyler F 2017 Virus/host cell crosstalk in hypoxic HPV-positive cancer cells. Viruses 9:174. doi:10.3390/v9070174. PMC5537666 28678198

[15] Manning BD, Toker A 2017 AKT/PKB signaling: navigating the network. Cell 169:381–405. doi:10.1016/j.cell.2017.04.001. 28431241 PMC5546324

[16] Janku F, Yap TA, Meric-Bernstam F 2018 Targeting the PI3K pathway in cancer: are we making headway? Nat Rev Clin Oncol 15:273–291. doi:10.1038/nrclinonc.2018.28. 29508857

[17] Hou MM, Liu X, Wheler J, Naing A, Hong D, Coleman RL, Tsimberidou A, Janku F, Zinner R, Lu K, Kurzrock R, Fu S 2014 Targeted PI3K/AKT/mTOR therapy for metastatic carcinomas of the cervix: A phase I clinical experience. Oncotarget 5:11168–11179. doi:10.18632/oncotarget.2584. 25426553 PMC4294378

[18] Janku F, Hong DS, Fu S, Piha-Paul SA, Naing A, Falchook GS, Tsimberidou AM, Stepanek VM, Moulder SL, Lee JJ, Luthra R, Zinner RG, Broaddus RR, Wheler JJ, Kurzrock R 2014 Assessing PIK3CA and PTEN in early-phase trials with PI3K/AKT/mTOR inhibitors. Cell Rep 6:377–387. doi:10.1016/j.celrep.2013.12.035. 24440717 PMC4409143

[19] Juric D, Rodon J, Tabernero J, Janku F, Burris HA, Schellens JHM, Middleton MR, Berlin J, Schuler M, Gil-Martin M, Rugo HS, Seggewiss-Bernhardt R, Huang A, Bootle D, Demanse D, Blumenstein L, Coughlin C, Quadt C, Baselga J 2018 Phosphatidylinositol 3-kinase alpha-selective inhibition with alpelisib (BYL719) in PIK3CA-altered solid tumors: results from the first-in-human study. J Clin Oncol 36:1291–1299. doi:10.1200/JCO.2017.72.7107. 29401002 PMC5920739

[20] Zundel W, Schindler C, Haas-Kogan D, Koong A, Kaper F, Chen E, Gottschalk AR, Ryan HE, Johnson RS, Jefferson AB, Stokoe D, Giaccia AJ 2000 Loss of PTEN facilitates HIF-1-mediated gene expression. Genes Dev 14:391–396. 10691731 PMC316386

[21] Beitner-Johnson D, Rust RT, Hsieh TC, Millhorn DE 2001 Hypoxia activates Akt and induces phosphorylation of GSK-3 in PC12 cells. Cell Signal 13:23–27. 11257444 10.1016/s0898-6568(00)00128-5

[22] Stegeman H, Kaanders JH, Wheeler DL, van der Kogel AJ, Verheijen MM, Waaijer SJ, Iida M, Grenman R, Span PN, Bussink J 2012 Activation of AKT by hypoxia: a potential target for hypoxic tumors of the head and neck. BMC Cancer 12:463. doi:10.1186/1471-2407-12-463. 23046567 PMC3517352

[23] Cavadas MA, Mesnieres M, Crifo B, Manresa MC, Selfridge AC, Keogh CE, Fabian Z, Scholz CC, Nolan KA, Rocha LM, Tambuwala MM, Brown S, Wdowicz A, Corbett D, Murphy KJ, Godson C, Cummins EP, Taylor CT, Cheong A 2016 REST is a hypoxia-responsive transcriptional repressor. Sci Rep 6:31355. doi:10.1038/srep31355. 27531581 PMC4987654

[24] Chai TF, Leck YC, He H, Yu FX, Luo Y, Hagen T 2011 Hypoxia-inducible factor independent down-regulation of thioredoxin-interacting protein in hypoxia. FEBS Lett 585:492–498. doi:10.1016/j.febslet.2010.12.033. 21192937

[25] Havula E, Hietakangas V 2018 Sugar sensing by ChREBP/Mondo-Mlx-new insight into downstream regulatory networks and integration of nutrient-derived signals. Curr Opin Cell Biol 51:89–96. doi:10.1016/j.ceb.2017.12.007. 29278834

[26] Calleja V, Laguerre M, Parker PJ, Larijani B 2009 Role of a novel PH-kinase domain interface in PKB/Akt regulation: structural mechanism for allosteric inhibition. PLoS Biol 7:e17. doi:10.1371/journal.pbio.1000017. 19166270 PMC2628406

[27] Maira S-M, Stauffer F, Schnell C, García-Echeverría C 2009 PI3K inhibitors for cancer treatment: where do we stand? Biochem Soc Trans 37:265–272. doi:10.1042/BST0370265. 19143644

[28] Brunn GJ, Williams J, Sabers C, Wiederrecht G, Lawrence JC Jr, Abraham RT 1996 Direct inhibition of the signaling functions of the mammalian target of rapamycin by the phosphoinositide 3-kinase inhibitors, wortmannin and LY294002. EMBO J 15:5256–5267. doi:10.1002/j.1460-2075.1996.tb00911.x. 8895571 PMC452270

[29] Gharbi SI, Zvelebil MJ, Shuttleworth SJ, Hancox T, Saghir N, Timms JF, Waterfield MD 2007 Exploring the specificity of the PI3K family inhibitor LY294002. Biochem J 404:15–21. doi:10.1042/BJ20061489. 17302559 PMC1868829

[30] Dittmann A, Werner T, Chung CW, Savitski MM, Falth Savitski M, Grandi P, Hopf C, Lindon M, Neubauer G, Prinjha RK, Bantscheff M, Drewes G 2014 The commonly used PI3-kinase probe LY294002 is an inhibitor of BET bromodomains. ACS Chem Biol 9:495–502. doi:10.1021/cb400789e. 24533473

[31] Folkes AJ, Ahmadi K, Alderton WK, Alix S, Baker SJ, Box G, Chuckowree IS, Clarke PA, Depledge P, Eccles SA, Friedman LS, Hayes A, Hancox TC, Kugendradas A, Lensun L, Moore P, Olivero AG, Pang J, Patel S, Pergl-Wilson GH, Raynaud FI, Robson A, Saghir N, Salphati L, Sohal S, Ultsch MH, Valenti M, Wallweber HJ, Wan NC, Wiesmann C, Workman P, Zhyvoloup A, Zvelebil MJ, Shuttleworth SJ 2008 The identification of 2-(1H-indazol-4-yl)-6-(4-methanesulfonyl-piperazin-1-ylmethyl)-4-morpholin-4-yl-thieno[3,2-d]pyrimidine (GDC-0941) as a potent, selective, orally bioavailable inhibitor of class I PI3 kinase for the treatment of cancer. J Med Chem 51:5522–5532. doi:10.1021/jm800295d. 18754654

[32] Rohlfs M, Winkenbach S, Meyer S, Rupp T, Durst M 1991 Viral transcription in human keratinocyte cell lines immortalized by human papillomavirus type-16. Virology 183:331–342. 1647072 10.1016/0042-6822(91)90146-3

[33] Jonasch E, Hasanov E, Corn PG, Moss T, Shaw KR, Stovall S, Marcott V, Gan B, Bird S, Wang X, Do KA, Altamirano PF, Zurita AJ, Doyle LA, Lara PN Jr, Tannir NM 2017 A randomized phase 2 study of MK-2206 versus everolimus in refractory renal cell carcinoma. Ann Oncol 28:804–808. doi:10.1093/annonc/mdw676. 28049139 PMC5834088

[34] Pitz MW, Eisenhauer EA, MacNeil MV, Thiessen B, Easaw JC, Macdonald DR, Eisenstat DD, Kakumanu AS, Salim M, Chalchal H, Squire J, Tsao MS, Kamel-Reid S, Banerji S, Tu D, Powers J, Hausman DF, Mason WP 2015 Phase II study of PX-866 in recurrent glioblastoma. Neuro Oncol 17:1270–1274. doi:10.1093/neuonc/nou365. 25605819 PMC4588751

[35] Sarbassov DD, Guertin DA, Ali SM, Sabatini DM 2005 Phosphorylation and regulation of Akt/PKB by the rictor-mTOR complex. Science 307:1098–1101. doi:10.1126/science.1106148. 15718470

[36] Sarbassov DD, Ali SM, Kim DH, Guertin DA, Latek RR, Erdjument-Bromage H, Tempst P, Sabatini DM 2004 Rictor, a novel binding partner of mTOR, defines a rapamycin-insensitive and raptor-independent pathway that regulates the cytoskeleton. Curr Biol 14:1296–1302. doi:10.1016/j.cub.2004.06.054. 15268862

[37] Choo AY, Yoon SO, Kim SG, Roux PP, Blenis J 2008 Rapamycin differentially inhibits S6Ks and 4E-BP1 to mediate cell-type-specific repression of mRNA translation. Proc Natl Acad Sci U S A 105:17414–17419. doi:10.1073/pnas.0809136105. 18955708 PMC2582304

[38] Hirai H, Sootome H, Nakatsuru Y, Miyama K, Taguchi S, Tsujioka K, Ueno Y, Hatch H, Majumder PK, Pan BS, Kotani H 2010 MK-2206, an allosteric Akt inhibitor, enhances antitumor efficacy by standard chemotherapeutic agents or molecular targeted drugs in vitro and in vivo. Mol Cancer Ther 9:1956–1967. doi:10.1158/1535-7163.MCT-09-1012. 20571069

[39] Lindsley CW, Zhao Z, Leister WH, Robinson RG, Barnett SF, Defeo-Jones D, Jones RE, Hartman GD, Huff JR, Huber HE, Duggan ME 2005 Allosteric Akt (PKB) inhibitors: discovery and SAR of isozyme selective inhibitors. Bioorg Med Chem Lett 15:761–764. doi:10.1016/j.bmcl.2004.11.011. 15664853

[40] Gonzalez E, McGraw TE 2009 The Akt kinases: isoform specificity in metabolism and cancer. Cell Cycle 8:2502–2508. doi:10.4161/cc.8.16.9335. 19597332 PMC2997486

[41] Madhunapantula SV, Robertson GP 2017 Targeting protein kinase-b3 (akt3) signaling in melanoma. Expert Opin Ther Targets 21:273–290. doi:10.1080/14728222.2017.1279147. 28064546

[42] Kohn AD, Takeuchi F, Roth RA 1996 Akt, a pleckstrin homology domain containing kinase, is activated primarily by phosphorylation. J Biol Chem 271:21920–21926. 8702995 10.1074/jbc.271.36.21920

[43] Calleja V, Alcor D, Laguerre M, Park J, Vojnovic B, Hemmings BA, Downward J, Parker PJ, Larijani B 2007 Intramolecular and intermolecular interactions of protein kinase B define its activation in vivo. PLoS Biol 5:e95. doi:10.1371/journal.pbio.0050095. 17407381 PMC1845162

[44] Cross DA, Alessi DR, Cohen P, Andjelkovich M, Hemmings BA 1995 Inhibition of glycogen synthase kinase-3 by insulin mediated by protein kinase B. Nature 378:785–789. doi:10.1038/378785a0. 8524413

[45] Butz K, Hoppe-Seyler F 1993 Transcriptional control of human papillomavirus (HPV) oncogene expression: composition of the HPV type 18 upstream regulatory region. J Virol 67:6476–6486. 8411351 10.1128/jvi.67.11.6476-6486.1993 PMC238084

[46] Zhong H, Simons JW 1999 Direct comparison of GAPDH, beta-actin, cyclophilin, and 28S rRNA as internal standards for quantifying RNA levels under hypoxia. Biochem Biophys Res Commun 259:523–526. doi:10.1006/bbrc.1999.0815. 10364451

[47] Baker CC, Phelps WC, Lindgren V, Braun MJ, Gonda MA, Howley PM 1987 Structural and transcriptional analysis of human papillomavirus type 16 sequences in cervical carcinoma cell lines. J Virol 61:962–971. 3029430 10.1128/jvi.61.4.962-971.1987 PMC254051

[48] Günther T, Grundhoff A 2010 The epigenetic landscape of latent Kaposi sarcoma-associated herpesvirus genomes. PLoS Pathog 6:e1000935. doi:10.1371/journal.ppat.1000935. 20532208 PMC2880564

[49] Park IS, Chang X, Loyo M, Wu G, Chuang A, Kim MS, Chae YK, Lyford-Pike S, Westra WH, Saunders JR, Sidransky D, Pai SI 2011 Characterization of the methylation patterns in human papillomavirus type 16 viral DNA in head and neck cancers. Cancer Prev Res (Phila) 4:207–217. doi:10.1158/1940-6207.CAPR-10-0147. 21292634 PMC3079312

[50] Kooistra SM, Helin K 2012 Molecular mechanisms and potential functions of histone demethylases. Nat Rev Mol Cell Biol 13:297–311. doi:10.1038/nrm3327. 22473470

[51] Torigoe Y, Takahashi N, Hara M, Yoshimatsu H, Saikawa T 2009 Adrenomedullin improves cardiac expression of heat-shock protein 72 and tolerance against ischemia/reperfusion injury in insulin-resistant rats. Endocrinology 150:1450–1455. doi:10.1210/en.2008-1052. 19008310

[52] He XH, Li JJ, Xie YH, Tang YT, Yao GF, Qin WX, Wan DF, Gu JR 2004 Altered gene expression profiles of NIH3T3 cells regulated by human lung cancer associated gene CT120. Cell Res 14:487–496. doi:10.1038/sj.cr.7290252. 15625016

[53] Datta J, Majumder S, Kutay H, Motiwala T, Frankel W, Costa R, Cha HC, MacDougald OA, Jacob ST, Ghoshal K 2007 Metallothionein expression is suppressed in primary human hepatocellular carcinomas and is mediated through inactivation of CCAAT/enhancer binding protein alpha by phosphatidylinositol 3-kinase signaling cascade. Cancer Res 67:2736–2746. doi:10.1158/0008-5472.CAN-06-4433. 17363595 PMC2276570

[54] Pourreyron C, Reilly L, Proby C, Panteleyev A, Fleming C, McLean K, South AP, Foerster J 2012 Wnt5a is strongly expressed at the leading edge in non-melanoma skin cancer, forming active gradients, while canonical Wnt signalling is repressed. PLoS One 7:e31827. doi:10.1371/journal.pone.0031827. 22384081 PMC3285195

[55] Niehrs C 2006 Function and biological roles of the Dickkopf family of Wnt modulators. Oncogene 25:7469. doi:10.1038/sj.onc.1210054. 17143291

[56] Cho H, Mu J, Kim JK, Thorvaldsen JL, Chu Q, Crenshaw EB III, Kaestner KH, Bartolomei MS, Shulman GI, Birnbaum MJ 2001 Insulin resistance and a diabetes mellitus-like syndrome in mice lacking the protein kinase Akt2 (PKB beta). Science 292:1728–1731. doi:10.1126/science.292.5522.1728. 11387480

[57] Cho H, Thorvaldsen JL, Chu Q, Feng F, Birnbaum MJ 2001 Akt1/PKBalpha is required for normal growth but dispensable for maintenance of glucose homeostasis in mice. J Biol Chem 276:38349–38352. doi:10.1074/jbc.C100462200. 11533044

[58] Dummler B, Hemmings BA 2007 Physiological roles of PKB/Akt isoforms in development and disease. Biochem Soc Trans 35:231–235. doi:10.1042/BST0350231. 17371246

[59] Guo J, Chakraborty AA, Liu P, Gan W, Zheng X, Inuzuka H, Wang B, Zhang J, Zhang L, Yuan M, Novak J, Cheng JQ, Toker A, Signoretti S, Zhang Q, Asara JM, Kaelin WG Jr, Wei W 2016 pVHL suppresses kinase activity of Akt in a proline-hydroxylation-dependent manner. Science 353:929–932. doi:10.1126/science.aad5755. 27563096 PMC5326551

[60] Chang S, Park B, Choi K, Moon Y, Lee HY, Park H 2016 Hypoxic reprograming of H3K27me3 and H3K4me3 at the INK4A locus. FEBS Lett 590:3407–3415. doi:10.1002/1873-3468.12375. 27545759

[61] Adriaens ME, Prickaerts P, Chan-Seng-Yue M, van den Beucken T, Dahlmans VEH, Eijssen LM, Beck T, Wouters BG, Voncken JW, Evelo CTA 2016 Quantitative analysis of ChIP-seq data uncovers dynamic and sustained H3K4me3 and H3K27me3 modulation in cancer cells under hypoxia. Epigenetics Chromatin 9:48. doi:10.1186/s13072-016-0090-4. 27822313 PMC5090954

[62] Prickaerts P, Adriaens ME, Beucken TVD, Koch E, Dubois L, Dahlmans VEH, Gits C, Evelo CTA, Chan-Seng-Yue M, Wouters BG, Voncken JW 2016 Hypoxia increases genome-wide bivalent epigenetic marking by specific gain of H3K27me3. Epigenetics Chromatin 9:46. doi:10.1186/s13072-016-0086-0. 27800026 PMC5080723

[63] Bernard HU 2013 Regulatory elements in the viral genome. Virology 445:197–204. doi:10.1016/j.virol.2013.04.035. 23725692

[64] Adini I, Rabinovitz I, Sun JF, Prendergast GC, Benjamin LE 2003 RhoB controls Akt trafficking and stage-specific survival of endothelial cells during vascular development. Genes Dev 17:2721–2732. doi:10.1101/gad.1134603. 14597666 PMC280621

[65] Kazerounian S, Gerald D, Huang M, Chin YR, Udayakumar D, Zheng N, O'Donnell RK, Perruzzi C, Mangiante L, Pourat J, Phung TL, Bravo-Nuevo A, Shechter S, McNamara S, Duhadaway JB, Kocher ON, Brown LF, Toker A, Prendergast GC, Benjamin LE 2013 RhoB differentially controls Akt function in tumor cells and stromal endothelial cells during breast tumorigenesis. Cancer Res 73:50–61. doi:10.1158/0008-5472.CAN-11-3055. 23135917 PMC4201498

[66] Hettinger K, Vikhanskaya F, Poh MK, Lee MK, de Belle I, Zhang JT, Reddy SA, Sabapathy K 2007 c-Jun promotes cellular survival by suppression of PTEN. Cell Death Differ 14:218–229. doi:10.1038/sj.cdd.4401946. 16676006

[67] Chen C, Okayama H 1987 High-efficiency transformation of mammalian cells by plasmid DNA. Mol Cell Biol 7:2745–2752. 3670292 10.1128/mcb.7.8.2745-2752.1987 PMC367891

[68] Brummelkamp TR, Bernards R, Agami R 2002 A system for stable expression of short interfering RNAs in mammalian cells. Science 296:550–553. doi:10.1126/science.1068999. 11910072

[69] Sanjana NE, Shalem O, Zhang F 2014 Improved vectors and genome-wide libraries for CRISPR screening. Nat Methods 11:783–784. doi:10.1038/nmeth.3047. 25075903 PMC4486245

[70] Tuttle RL, Gill NS, Pugh W, Lee JP, Koeberlein B, Furth EE, Polonsky KS, Naji A, Birnbaum MJ 2001 Regulation of pancreatic beta-cell growth and survival by the serine/threonine protein kinase Akt1/PKBalpha. Nat Med 7:1133–1137. doi:10.1038/nm1001-1133. 11590437

[71] Lu S, Gu X, Hoestje S, Epner DE 2002 Identification of an additional hypoxia responsive element in the glyceraldehyde-3-phosphate dehydrogenase gene promoter. Biochim Biophys Acta 1574:152–156. 11955624 10.1016/s0167-4781(01)00359-1

[72] Leitz J, Reuschenbach M, Lohrey C, Honegger A, Accardi R, Tommasino M, Llano M, von Knebel Doeberitz M, Hoppe-Seyler K, Hoppe-Seyler F 2014 Oncogenic human papillomaviruses activate the tumor-associated lens epithelial-derived growth factor (LEDGF) gene. PLoS Pathog 10:e1003957. doi:10.1371/journal.ppat.1003957. 24604027 PMC3946365

[73] Honegger A, Schilling D, Bastian S, Sponagel J, Kuryshev V, Sultmann H, Scheffner M, Hoppe-Seyler K, Hoppe-Seyler F 2015 Dependence of intracellular and exosomal microRNAs on viral E6/E7 oncogene expression in HPV-positive tumor cells. PLoS Pathog 11:e1004712. doi:10.1371/journal.ppat.1004712. 25760330 PMC4356518

[74] Feoktistova M, Geserick P, Leverkus M 2016 Crystal violet assay for determining viability of cultured cells. Cold Spring Harb Protoc 2016:pdb.prot087379. doi:10.1101/pdb.prot087379. 27037069

[75] Thompson A, Schafer J, Kuhn K, Kienle S, Schwarz J, Schmidt G, Neumann T, Johnstone R, Mohammed AK, Hamon C 2003 Tandem mass tags: a novel quantification strategy for comparative analysis of complex protein mixtures by MS/MS. Anal Chem 75:1895–1904. 12713048 10.1021/ac0262560

[76] Cole ST, Danos O 1987 Nucleotide sequence and comparative analysis of the human papillomavirus type 18 genome. Phylogeny of papillomaviruses and repeated structure of the E6 and E7 gene products. J Mol Biol 193:599–608. 3039146 10.1016/0022-2836(87)90343-3

[77] Brugarolas J, Lei K, Hurley RL, Manning BD, Reiling JH, Hafen E, Witters LA, Ellisen LW, Kaelin WG Jr. 2004 Regulation of mTOR function in response to hypoxia by REDD1 and the TSC1/TSC2 tumor suppressor complex. Genes Dev 18:2893–2904. doi:10.1101/gad.1256804. 15545625 PMC534650

