# Supplementary figures and images for: Repression of Human Papillomavirus Oncogene Expression under Hypoxia Is Mediated by PI3K/mTORC2/AKT Signaling
Source: mBio. 2019 Feb 12;10(1):e02323-18. doi: 10.1128/mBio.02323-18 (PMC6372795; doi:10.1128/mBio.02323-18)

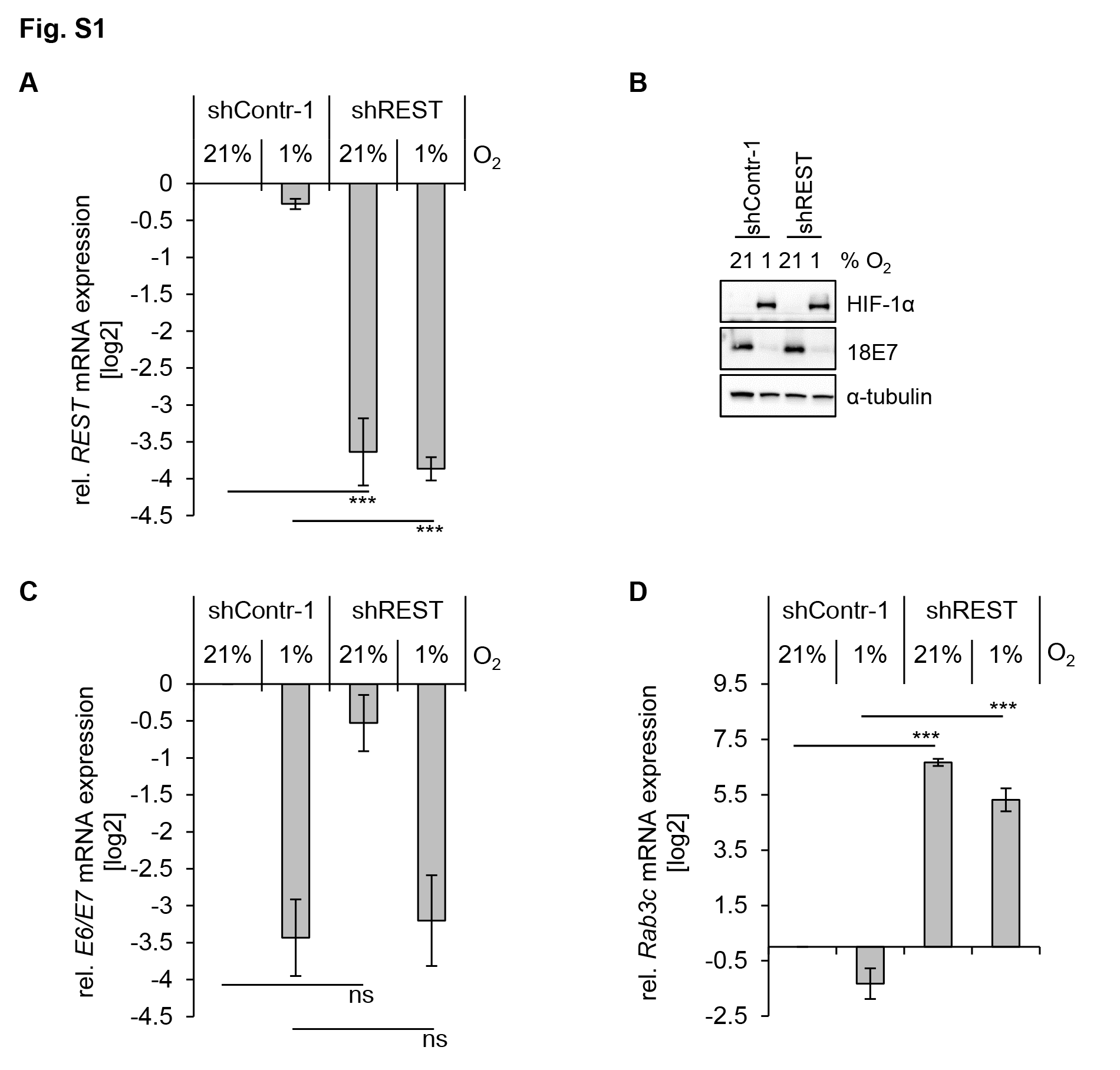

Supplement: FIG S1 [file mBio.02323-18-sf001.tif]

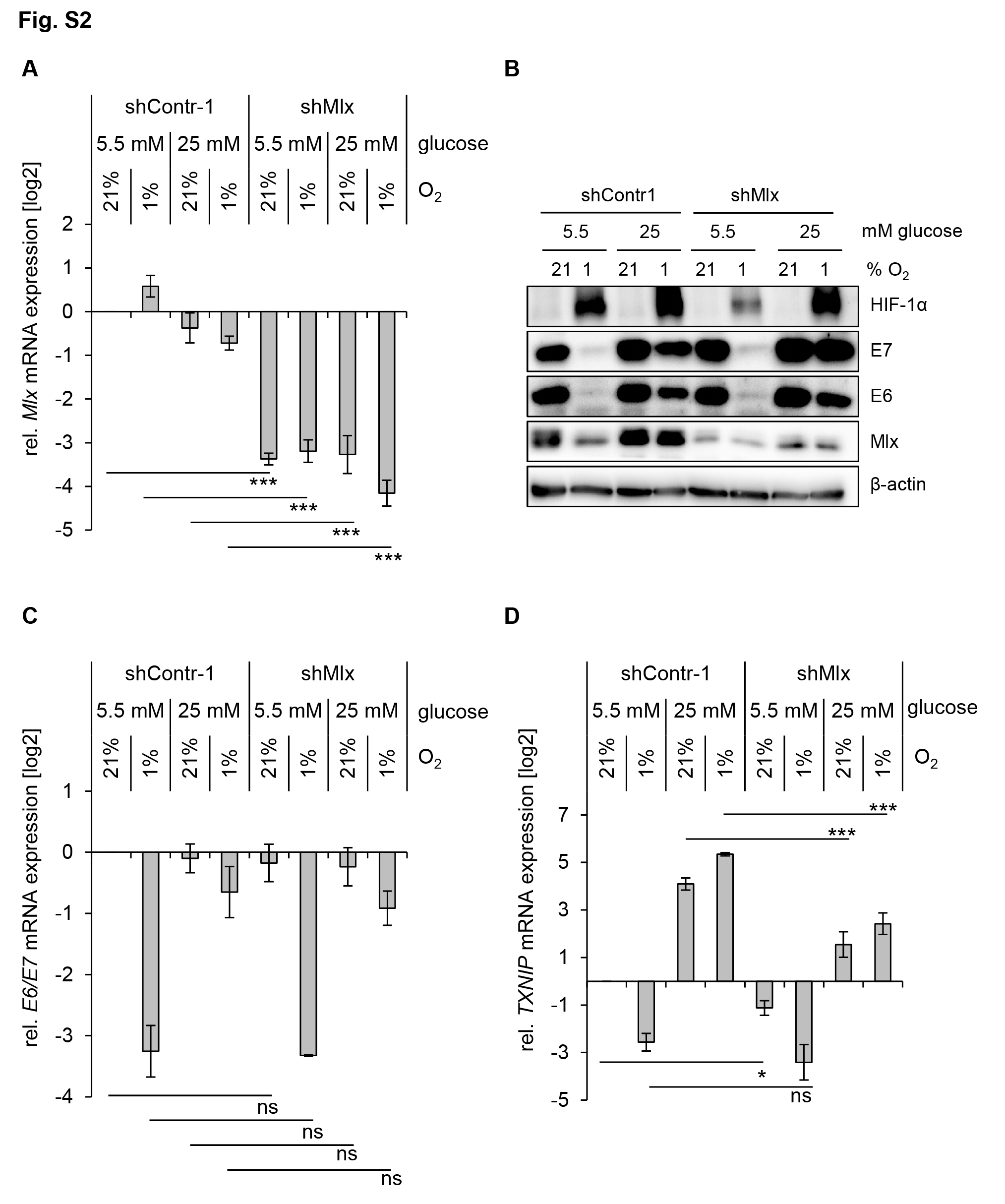

Supplement: FIG S2 [file mBio.02323-18-sf002.tif]

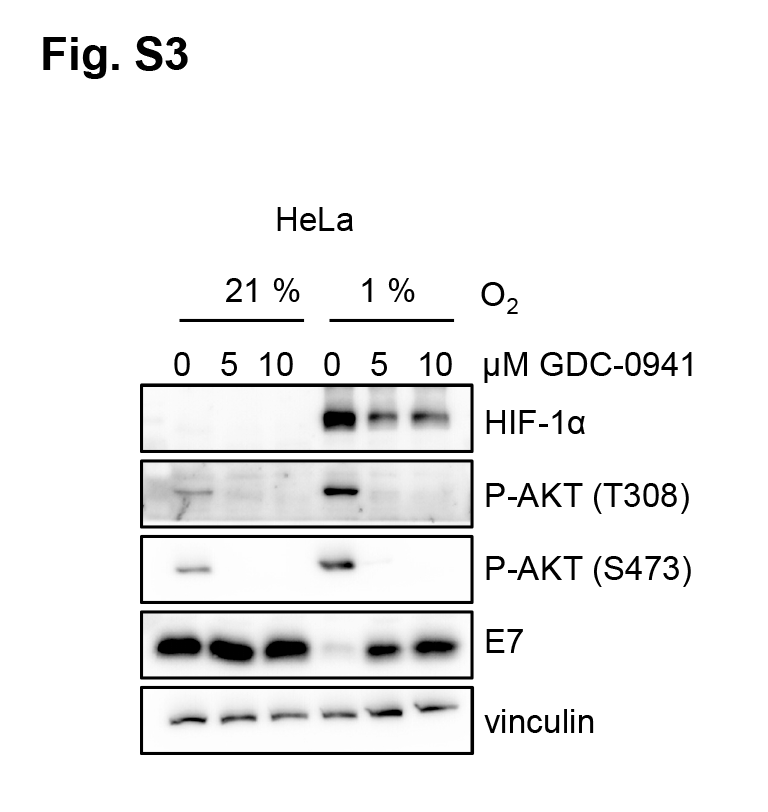

Supplement: FIG S3 [file mBio.02323-18-sf003.tif]

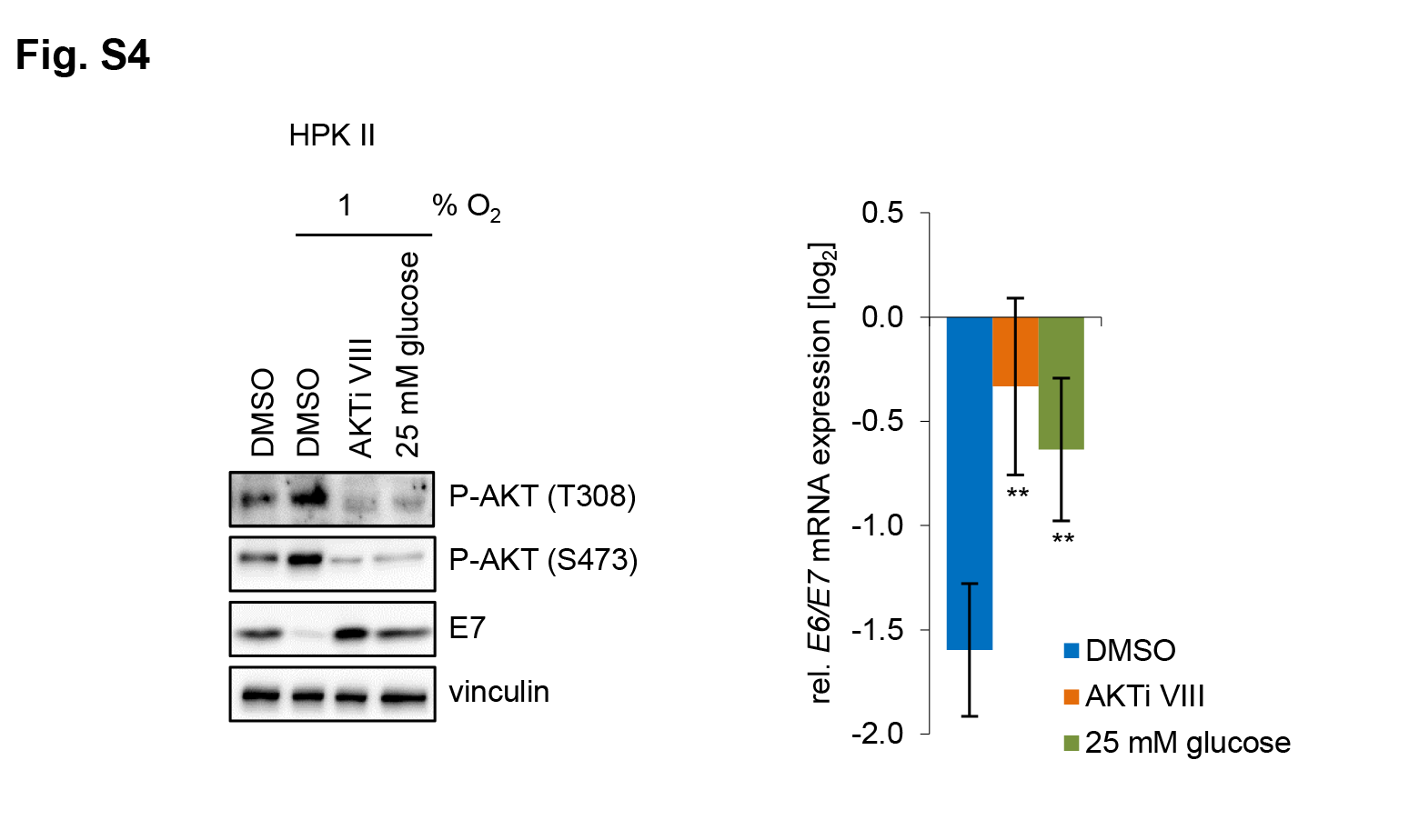

Supplement: FIG S4 [file mBio.02323-18-sf004.tif]

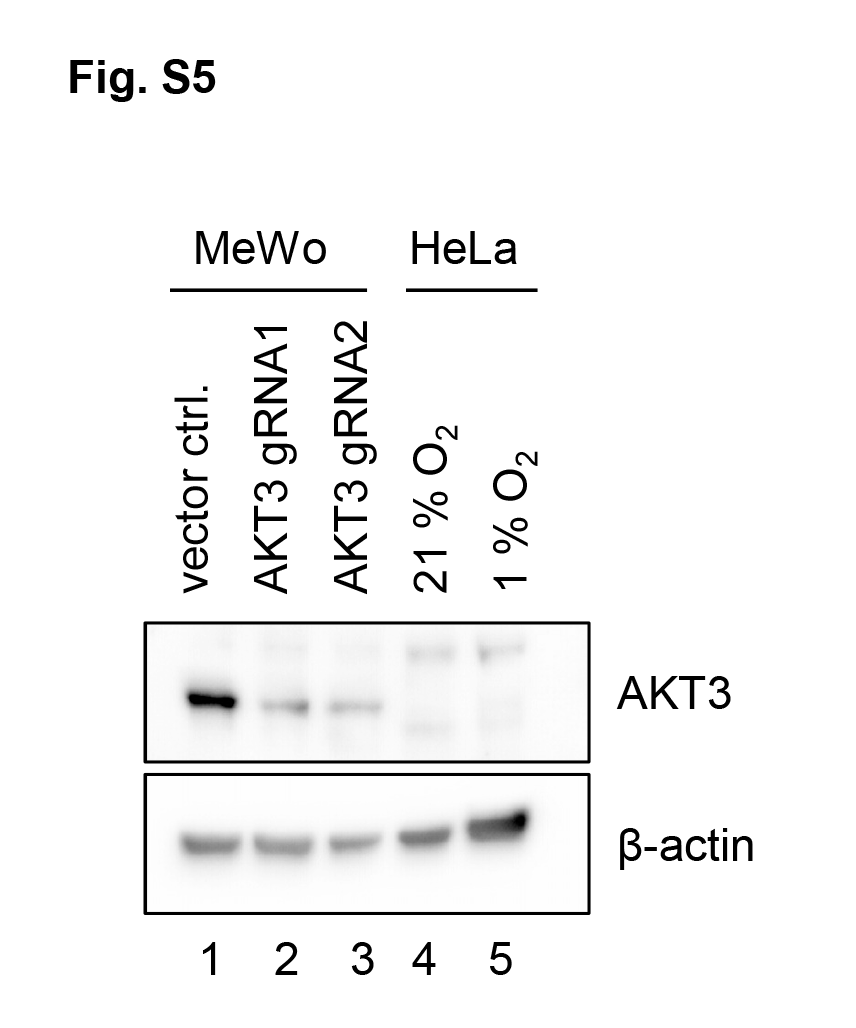

Supplement: FIG S5 [file mBio.02323-18-sf005.tif]

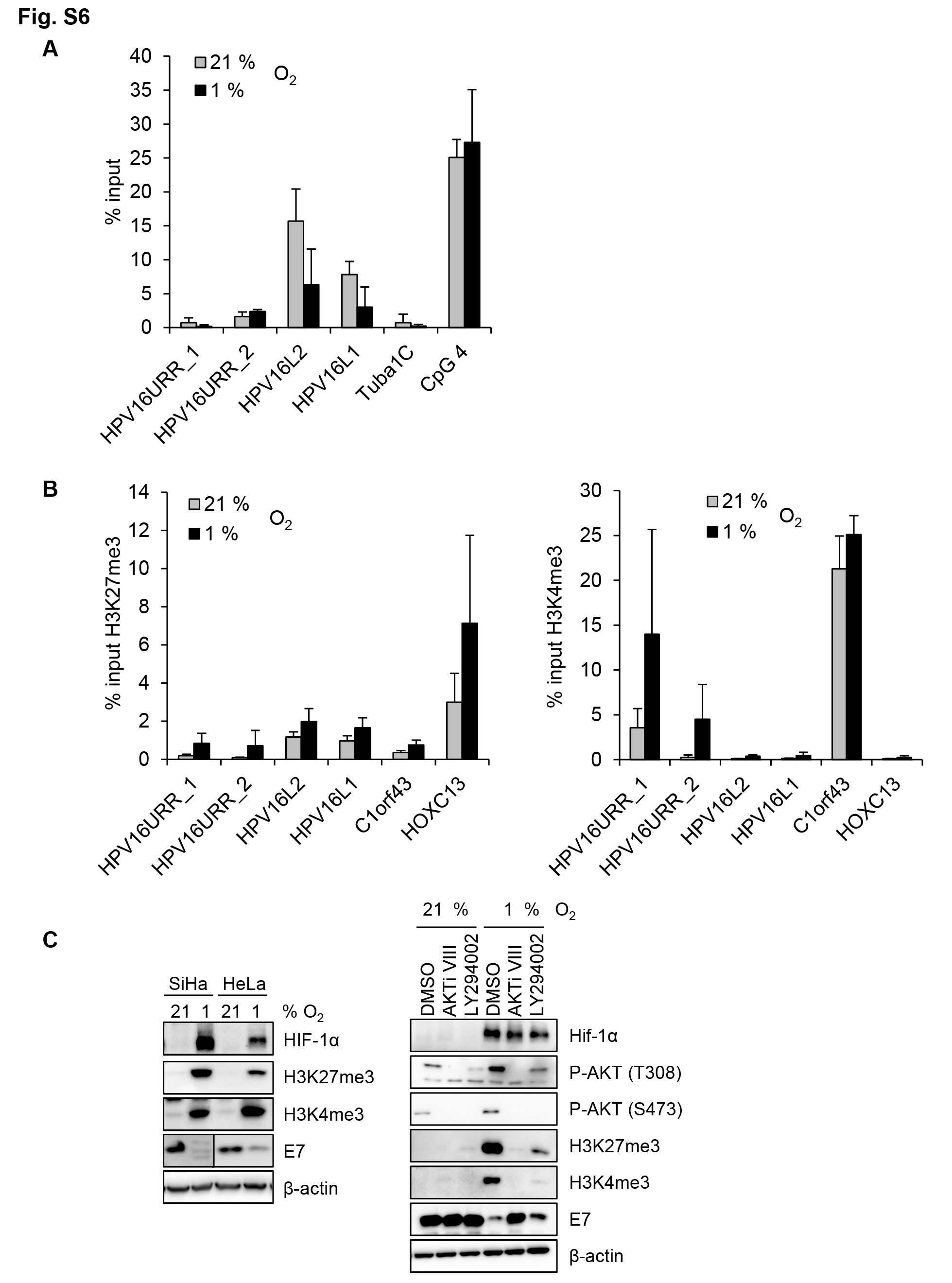

Supplement: FIG S6 [file mBio.02323-18-sf006.tif]

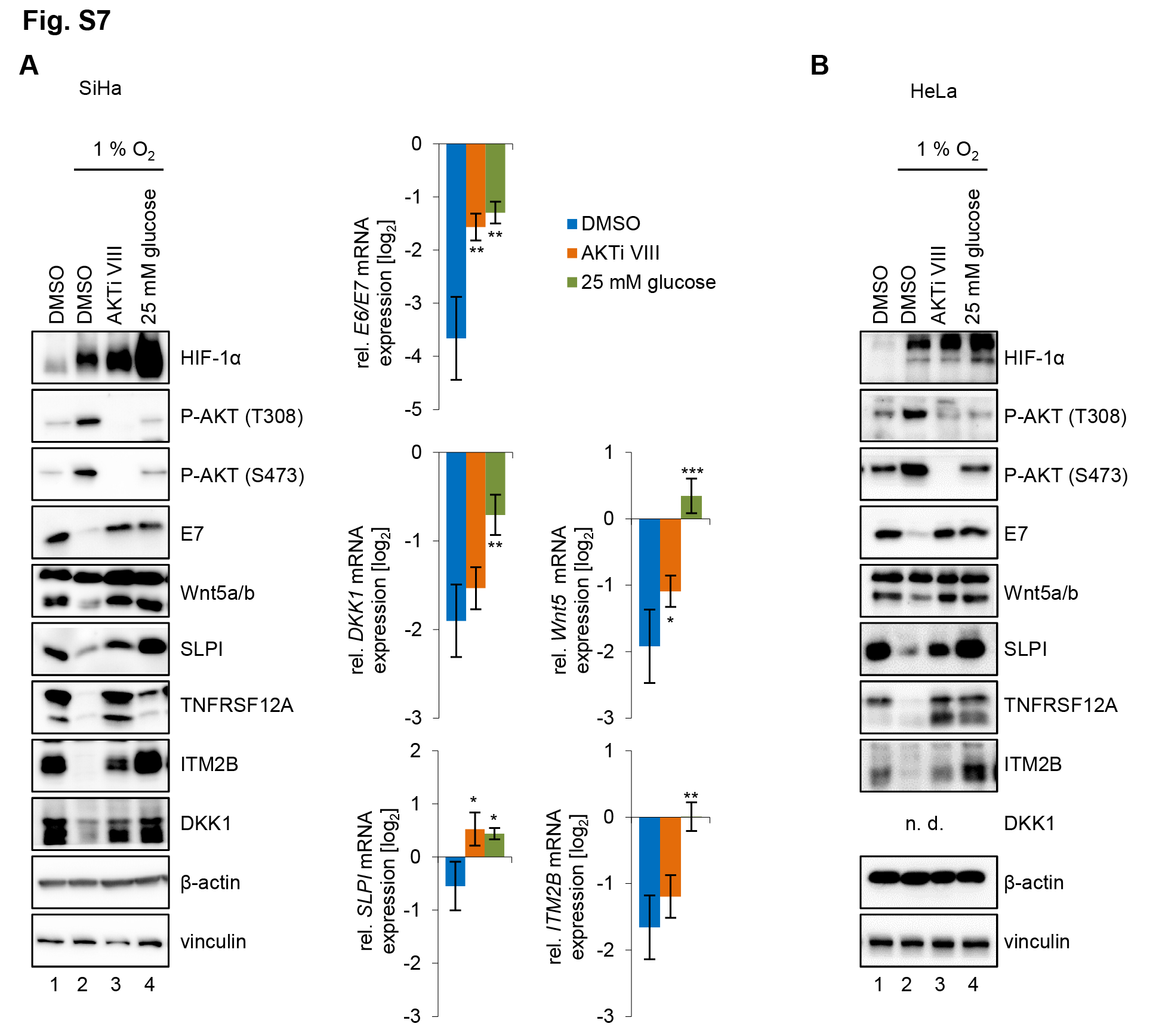

Supplement: FIG S7 [file mBio.02323-18-sf007.tif]
