# Supplementary material for: Repression of Human Papillomavirus Oncogene Expression under Hypoxia Is Mediated by PI3K/mTORC2/AKT Signaling
Source: mBio. 2019 Feb 12;10(1):e02323-18. doi: 10.1128/mBio.02323-18 (PMC6372795; doi:10.1128/mBio.02323-18)
Supplement: TEXT S1 [file mBio.02323-18-s0001.docx]

# Supplemental Methods

# Immunoblot analyses

All immunoblot analyses were performed at least thrice. The following antibodies were employed: anti–β-actin (A2228; Sigma-Aldrich), anti-HPV18 E6 (AVC 399) and anti-HPV16 E6 (AVC 843, kind gift from Johannes Schweizer) (Arbor Vita Corporation), anti-HPV18 E7 (E7C), anti-HPV16 E7 (NM2, kind gift from Martin Müller, German Cancer Research Center), anti–HIF-1α (610959, BD Biosciences), anti-AKT (#9272), anti-phospho-AKT (S473) (#4058), anti-phospho-AKT (T308) (#9275), anti–phospho-S6 (S235/236) (#2211), anti–4E-BP1 (#9452), anti–phospho-4E-BP1 (S65) (#9451), anti-p70S6K (#9202), anti–phospho-p70S6K (T389) (#9234), anti-phospho-GSK-3-α/β (S21/S9) (#9331), anti-Rictor (#2114), anti-H3K4me3 (#9751), anti-H3K27me3 (#9733), anti-Mlx (#85570), anti-TNFRSF12A (#4403), anti-Wnt5a/b (#2530) (Cell Signaling Technology), anti-AKT2 (sc-5270), anti-DKK1 (sc-374574), anti-ITM2B (sc-374362), anti-SLPI (sc-373802), anti-vinculin (sc-73614) (Santa Cruz Biotechnology), anti-AKT3 (GMA104, 05-780, Upstate), anti-α-tubulin (Merck). Secondary antibodies were as follows: anti-chicken IgG (sc-2428), anti-mouse IgG (sc-2005), anti-rabbit IgG (sc-2004) (Santa Cruz Biotechnology).

# RNA extraction and qRT-PCR

RNA was isolated from cells using the PureLink RNA Mini Kit (Ambion, Life Technologies). cDNA was synthesized from 0.5 µg of RNA with the the ProtoScript® II First Strand cDNA Synthesis Kit (NEB) using random primers. Primer sequences were as follows: DKK1 for: 5’-TGGAACTCCCCTGTGATTGC-3’, DKK1 rev: 5’- AATAGGCAGTGCAGCACCTT-3’, HPV18 E6/E7 for: 5’-ATGCATGGACCTAAGGCAAC-3’, HPV18E6/E7 rev: 5’-AGGTCGTCTGCTGAGCTTTC-3’, ITM2B for: 5’-CAGTGTGCCTGTCCCAGAGT-3’, ITM2B rev: 5’-AGGCAAATAGGTTCCAGCCTTG-3’, REST for: 5’-ACTTTGTCCTTACTCAAGTTCTCA-3’, REST rev: 5’-TCTTGCATGGCGGGTTACTT-3’, Rab3c for: 5’-TCTGCATTCGTCAGCACAGT-3’, Rab3c rev: 5’-GCCCATGGCTCCACGATAAT-3’, Mlx for: 5’-GGTCCGGTGGGTACAAGATG-3’, Mlx rev: 5’-ACTGTCCTCATCATCGGGGT-3’, SLPI for: 5’-GATCCTGTTGACACCCCAA -3’, SLPI rev: 5’-CAAGTCTCAGGGTGGAAAGG-3’, TXNIP for: 5’-GGCGGGTGTCTGTCTCTGCT-3’, TXNIP rev: 5’-GGCAAGGTAAGTGTGGCGGG-3’, 18S rRNA for: 5’-CATGGCCGTTCTTAGTTGGT-3’, Wnt5a for: 5’-GCACCAGAGCAGACAACCTA-3’, Wnt5a rev: 5’- GGCCAGCATCACATCACAAC-3’, 18S rRNA rev: 5’-ATGCCAGAGTCTCGTTCGTT-3’. The HPV18 E6/E7 primers detect all three transcript classes coding for HPV18 E6 and E7 (1). All RNA measurements were performed independently at least thrice as duplicate values. Relative quantification was performed using the comparative Ct (2^−ΔΔCt^) method (2) with normalization to 18S rRNA as internal reference and relative to a calibrator sample. Data is presented following logarithmic transformation and analyzed for statistical significance by one-way ANOVA (*p<0.05, **p<0.01, ***p < 0.001).

# Methylated DNA-immunoprecipitation (MeDIP)

MeDIP analysis was essentially performed as described previously (3, 4). Briefly, genomic DNA was isolated by proteinase K digestion, phenol chloroform extraction and ethanol precipitation. Purified DNA (10µg, measured by Qubit) was diluted in 100 µl TE buffer and sheared by sonication using a Bioruptor NGS device (Diagenode) for 12 cycles (30 sec on and 30 sec off) to an average fragment size of 300 bp as judged by agarose gelelectrophoresis. A total of 5 µg sheared DNA was diluted in 500 µl TE buffer and denatured at 98 °C for 10 min, followed by incubation on ice for 10 min. 50 µl denatured DNA were saved as input sample. IPs were performed by adding 50 µl 10x IP-buffer (100 mM Na-phosphate buffer, pH 7.0, 1.4 mM NaCl, 0.5 % Triton X-100) and 2 µg of a 5´-methylcytidine specific antibody (*5-mc 33D3, #C15200006-100,* Diagenode) for 2 h on a rotating wheel at 4 °C. 50 µl magnetic Dynabeads® M-280 Sheep anti-Mouse IgG (Invitrogen) were prewashed twice in 1x IP-buffer and blocked with 1 mg/ml BSA in 1x IP buffer for 1 h at 4°C. Blocked beads were collected in 50 µl 1x IP buffer before adding them to the DNA-antibody complexes for 2 h at 4°. Samples were then washed five times for 10 min using TE buffer and collected in 200 µl TE buffer. Input DNA (50 µl) was diluted in 150 µl TE buffer and treated thereafter similar to precipitated samples. Contaminating RNA was degraded by incubation with 8 µl RNAseA (1 0mg/ml) for 30 min at 37 °C. DNA was eluted by addition of 200 µl elution buffer (50 mM Tris-HCl pH 8.0, 10 mM EDTA, 1 % SDS) supplemented with 7µl 300 mM CaCl_2_ and 4 µl ProteinaseK (20 mg/ml). Samples were incubated shaking at 65 °C for 30 min. DNA was purified twice by standard phenol-chloroform extraction and ethanol precipitation. Precipitated DNA was collected in 15 µl H_2_O and analyzed by qPCR (Rotorgene 6000, QIAgen; SensiMix™ SYBR® Hi-ROX, Bioline Cat#QT605-05) using the following forward (for) and reverse (rev) primers: HPV16URR_1 for: 5’-GTTGAACCGAAACCGGTTAGT-3’, HPPV16URR_1 rev: 5’-TCCTGAAACATTGCAGTTCTCTT-3’, HPV16URR_2 for: 5’-ACTGCTTGCCAACCATTCCA-3’, HPV16URR_2 rev: 5’-TAAGGCGTTGGCGCATAGTG-3’, HPV16L1 for: 5’-GTCGTGGTCAGCCATTAGGT-3’, HPV16L1 rev: 5’-TGCTGCATAAGCACTAGCATTT-3’, HPV16L2 for: 5’-GCGGACGCACTGGGTATATT-3’, HPV16L2 rev: 5’-AAGGGCCCACAGGATCTACT-3’, Tuba1C for: 5’-TGGTAGTCTGTTAGTGGGAGATCCT-3’, Tuba1C rev: 5’-GGTCTGCGGCGGTGAA-3’, CpG 4 for: 5’-GCTTTTCGAAGCTGTTGGAG-3’, CpG 4 rev: 5’-GGAAGATGGCGAATTCCTTT-3’. Tuba1C (tubulin alpha-1c chain) served as control for a non-methylated region and CpG 4 (CpG island in the SHANK1 gene) for a highly methylated region.

# Chromatin-immunoprecipitation (ChIP)

ChIP was performed essentially as described in detail previously (3, 5). Briefly, cells were crosslinked with 1 % formaldehyde for 10 min. Reactions were quenched by addition of glycine to a final concentration of 0.125 M. All following buffers contained 1x protease inhibitor cocktail (Roche) and 1 mM PMFS. Cells were incubated in 1 ml buffer 1 (50 mM Hepes-KOH, 140 mM NaCl, 1 mM EDTA, 10 % glycerol, 0.5 % NP-40, 0.25 % Triton X-100) for 10 min on ice to isolate nuclei. After centrifugation (1,350 x g 5 min), nuclei were washed with 1 ml buffer 2 (10 mM Tris-HCl, 200 mM NaCl, 1 mM EDTA, 0.5 mM EGTA). Pelleted nuclei were lysed in 1 ml buffer 3 (1 % SDS, 10 mM EDTA, 50 mM Tris-HCl) by multiple pipetting and chromatin was fragmented to approximately nucleosome size using a BioruptorTM (Diagenode). After addition of 100 µl 10% Triton X-100 Cell debris was pelleted (20,000 x g, 4°C) and chromatin containing supernatant was collected. Chromatin of 1x10^6^ cells was diluted 1:10 in dilution buffer (0.01 % SDS, 1.1 % Triton X-100, 1.2 mM EDTA, 16.7 mM Tris-HCl, 167 mM NaCl). 4 µg of the respective antibody (Rabbit monoclonal anti H3K4-me3 (clone MC315), Merck Millipore Cat#04-745; Rabbit polyclonal anti H3K27-me3, Merck Millipore Cat#07-449) was added to the sample and incubated for 16 h at 4°C rotating. 50 µl BSA-blocked ProteinG sepharose beads (GE Healthcare) were added to precipitate the chromatin-immunocomplexes and incubated for 1 h at 4 °C. Beads were washed once with 1 ml of the following buffers: low-salt buffer (0.1 % SDS, 1 % Triton X-100, 2 mM EDTA, 20 mM Tris-HCl, 150 mM NaCl); high-salt buffer (0.1 % SDS, 1 % Triton X-100, 2 mM EDTA, 20 mM Tris-HCl, 500 mM NaCl); LiCl-wash buffer (0.25 M LiCl, 1 % Nonidet P-40, 1 % Na-deoxycholate, 1 mM EDTA, 10 mM Tris-HCl). Subsequently, beads were washed twice with TE buffer omitting protease inhibitors. Chromatin was eluted from the beads by incubation in 210 µl SDS containing elution-buffer (50 mM Tris-HCl pH 8.0, 10 mM EDTA, 1 % SDS) for 30 min at 65°C and supernatant was transferred to a fresh tube. Input DNA samples were diluted in 200 µl elution buffer and treated thereafter similar to ChIP samples. After adding 8 µl of a 5 M NaCl stock solution, input and ChIP chromatin was de-crosslinked at 65 °C overnight. Contaminating RNA was degraded by addition of 200 µl TE buffer containing 8 µl RNAseA (10 mg/ml) for 2 h at 37 °C. Subsequently, 7 µl of CaCl_2_ solution (300 mM CaCl_2_ in 10 mM Tris-HCl) and 4 µl ProteinaseK (40 mg/ml) were added and incubated for 1 h at 55 °C to degrade proteins. DNA was purified by standard phenol-chloroform extraction and ethanol precipitation and recoverd in 55 µl 10 mM Tris-HCl. Precipitated DNA was analyzed by qPCR using the primers and qPCR setup as described for MeDIP. C1orf43 served as a positive control for an actively transcribed region and the HOXC13 gene as a positive control for a transcriptionally silenced region. Primer sequences were as follows: C1orf43 for: 5’-AGTGGGTGGAGAATGCAGAC-3’, C1orf43 rev: 5’-GAGATTACCCCACCCCATTC-3’, HOXC13 for: 5’-GAGCCCGAGATTCACTCAAC-3’, HOXC13 rev: 5’-TTATGCCCAGTTTTGGGGTA-3’.

# TMT mass spectrometry (MS) analyses

SiHa cells were treated with DMSO or 10 µM AKTi VIII, cultured in medium containing 5.5 or 25 mM glucose and incubated for 17 h at 21 % or 1 % O_2_. Cell pellets were suspended in 0.1 % (w/v) RapiGest-SF (Waters) in 100 mM triethylammonium bicarbonate (TEAB, pH 8). Cell lysis and shearing of chromatin was performed in a Bioruptor Pico (Diagenode) in a final volume of max. 300 µl, 15-20 cycles of 30’’/30’’ (ON/OFF) at 4°C. Protein concentration was determined by BCA-assay following the manufacturer’s protocol (Thermo Fisher Scientific). Prior to protein digestion disulfide bonds were reduced by 5 mM DTT for 30 min at 60 °C and subsequently alkylated with 15 mM 2‑chloroacetamide for 30 min at room temperature (RT). Proteolytic digestion was performed with trypsin (sequencing grade modified, Promega) in a protease-to-protein ratio of 1:50 (w/w) overnight at 37 °C, shaking at 700 rpm. Following digestion, 1 % trifluoroacetic acid was added (pH < 2) to stop digestion and break down RapiGest by incubation at 37 °C for 30 min followed by centrifugation at 20,000 x g for 10 min. Supernatants were transferred to a new tube and dried in a vacuum centrifuge.

Isobaric labeling of the peptides was performed with 10-plex TMT reagents according to the manufacturer’s protocol (Thermo Fisher Scientific). In brief, 10 µg of sample was dissolved in 10 µl, 100 mM TEAB, vortexed and incubated for 10 min at RT. TMT10-plex reagents (0.8 mg) were dissolved in 41 μl of acetonitrile (ACN, LC-MS grade). 4.1 µL of TMT reagent was combined with sample and incubated for 1 h at RT. The reaction was quenched by adding 8 μl of 5 % hydroxylamine and incubation for 15 min. Labeling efficacy was checked for each sample by MS prior to combining equal amounts and drying in a vacuum centrifuge prior to fractionation.

Dried samples were dissolved in 20 mM ammonium formate (pH 10), prior to fractionation on a 1200 Infinity HPLC system (Agilent) with a Gemini C18 column (3 µm, 110 Å, 100 × 1.0 mm; Phenomenex) using a linear 60 min gradient from 0 % to 35 % (v/v) ACN in 20 mM ammonium formate (pH 10) at a flow rate of 0.1 ml/min. Sixty 1-min fractions were collected and pooled into twelve fractions, dried and reconstituted in 0.1 % formic acid (FA). Fractions were injected by an Easy-nLC 1200 nano-UPLC (Thermo Fisher Scientific) onto a trap column (Pepmap, 100 μm x 2cm, C18, 5 μm 100Å pores) and separated on an analytical column (PepMap RSLC 75 μm × 50 cm, nanoViper, C18, 2 µM, 100Å) by applying a multistep gradient (Solvent A: 0.1 % FA in water, Solvent B: 0.1 % FA, 80 % ACN 29.9 % water) from : 3–50 % B over 90 min. Eluting peptides were electro-sprayed by applying 2 kV on a 10 µm Picotip coated emitter (New Objective) into an Orbitrap Fusion™ Tribrid™ (Thermo Fisher Scientific) mass spectrometer operated in data dependent mode of acquisition using the vendor supplied default settings for synchronous precursor selection (SPS) MS3 fragmentation (6).

The mass spectra were analyzed using Proteome Discoverer 2.1.0.81 (Thermo Fisher Scientific), using the SEQUEST search engine against UniProtKB/Swiss-Prot databases of Homo sapiens (21.06.2018), and human papillomavirus 16 (21.06.2018) proteome databases. Search settings: digestion reagent: trypsin, precursor and product ion tolerances were set at 20 ppm and 0.5 Da, respectively. Carbamidomethylation of cysteine was set as a fixed modification, oxidation of methionine as a variable modification. For TMT labels, TMT6-plex on lysine and peptide N-termini were set as a static modification. The percolator algorithm was used as the false discovery rate (FDR) calculator, and all peptides were filtered at a ‘strict’ target FDR level of 0.01. Proteins were tested for differential expression with Linear Models for Microarray Data (limma (7), version 3.36.2; R, version 3.5.1). Significant events (FDR < 0.05, |log FC| ≥ 1) were identified after adjusting for multiple testing according to Benjamini & Hochberg. The heatmap showing differential expression between hypoxia and normoxia was generated using the pandas (0.23.3) and seaborn (0.9.0) python packages. Hierarchical clustering was performed using scikit-learn, using Euclidean distance and the average linkage method. The mass spectrometry proteomics data have been deposited to the ProteomeXchange Consortium via the PRIDE (8) partner repository with the dataset identifier PXD011095.

# Supplemental References

1. Honegger A, Schilling D, Bastian S, Sponagel J, Kuryshev V, Sultmann H, Scheffner M, Hoppe-Seyler K, Hoppe-Seyler F. 2015. Dependence of intracellular and exosomal microRNAs on viral E6/E7 oncogene expression in HPV-positive tumor cells. PLoS Pathog 11:e1004712.

2. Livak KJ, Schmittgen TD. 2001. Analysis of relative gene expression data using real-time quantitative PCR and the 2(-Delta Delta C(T)) Method. Methods 25:402-8.

3. Günther T, Grundhoff A. 2010. The epigenetic landscape of latent Kaposi sarcoma-associated herpesvirus genomes. PLoS Pathog 6:e1000935.

4. Weber M, Davies JJ, Wittig D, Oakeley EJ, Haase M, Lam WL, Schubeler D. 2005. Chromosome-wide and promoter-specific analyses identify sites of differential DNA methylation in normal and transformed human cells. Nat Genet 37:853-62.

5. Günther T, Theiss JM, Fischer N, Grundhoff A. 2016. Investigation of Viral and Host Chromatin by ChIP-PCR or ChIP-Seq Analysis. Curr Protoc Microbiol 40:1e.10.1-21.

6. McAlister GC, Nusinow DP, Jedrychowski MP, Wuhr M, Huttlin EL, Erickson BK, Rad R, Haas W, Gygi SP. 2014. MultiNotch MS3 enables accurate, sensitive, and multiplexed detection of differential expression across cancer cell line proteomes. Anal Chem 86:7150-8.

7. Smyth GK. 2004. Linear models and empirical bayes methods for assessing differential expression in microarray experiments. Stat Appl Genet Mol Biol 3:Article3.

8. Vizcaino JA, Csordas A, del-Toro N, Dianes JA, Griss J, Lavidas I, Mayer G, Perez-Riverol Y, Reisinger F, Ternent T, Xu QW, Wang R, Hermjakob H. 2016. 2016 update of the PRIDE database and its related tools. Nucleic Acids Res 44:D447-56.
